# New ABA-Hypersensitive Arabidopsis Mutants Are Affected in Loci Mediating Responses to Water Deficit and *Dickeya dadantii* Infection

**DOI:** 10.1371/journal.pone.0020243

**Published:** 2011-05-25

**Authors:** Anne Plessis, Raphaël Cournol, Delphine Effroy, Viridiana Silva Pérez, Lucy Botran, Yvan Kraepiel, Anne Frey, Bruno Sotta, Gabriel Cornic, Jeffrey Leung, Jérôme Giraudat, Annie Marion-Poll, Helen M. North

**Affiliations:** 1 Institut Jean-Pierre Bourgin, UMR1318, INRA, AgroParisTech, Versailles, France; 2 Laboratoire des Interactions Plantes-Pathogènes, UMR217, AgroParisTech, INRA, UPMC Université Paris 6, Paris, France; 3 Laboratoire de Physiologie Cellulaire et Moléculaire des Plantes UR5, UPMC Université Paris 6, Paris, France; 4 Laboratoire d'Ecologie, Systématique et Evolution, UMR8079 IFR 87, Université Paris-Sud, CNRS, Orsay, France; 5 Institut des Sciences du Végétal, UPR2355, CNRS, Gif-sur-Yvette, France; USDA-ARS, United States of America

## Abstract

On water deficit, abscisic acid (ABA) induces stomata closure to reduce water loss by transpiration. To identify *Arabidopsis thaliana* mutants which transpire less on drought, infrared thermal imaging of leaf temperature has been used to screen for suppressors of an ABA-deficient mutant (*aba3-1*) cold-leaf phenotype. Three novel mutants, called *hot ABA-deficiency suppressor* (*has*), have been identified with hot-leaf phenotypes in the absence of the *aba3* mutation. The defective genes imparted no apparent modification to ABA production on water deficit, were inherited recessively and enhanced ABA responses indicating that the proteins encoded are negative regulators of ABA signalling. All three mutants showed ABA-hypersensitive stomata closure and inhibition of root elongation with little modification of growth and development in non-stressed conditions. The *has2* mutant also exhibited increased germination inhibition by ABA, while ABA-inducible gene expression was not modified on dehydration, indicating the mutated gene affects early ABA-signalling responses that do not modify transcript levels. In contrast, weak ABA-hypersensitivity relative to mutant developmental phenotypes suggests that *HAS3* regulates drought responses by both ABA-dependent and independent pathways. *has1* mutant phenotypes were only apparent on stress or ABA treatments, and included reduced water loss on rapid dehydration. The *HAS1* locus thus has the required characteristics for a targeted approach to improving resistance to water deficit. In contrast to *has2*, *has1* exhibited only minor changes in susceptibility to *Dickeya dadantii* despite similar ABA-hypersensitivity, indicating that crosstalk between ABA responses to this pathogen and drought stress can occur through more than one point in the signalling pathway.

## Introduction

Environmental abiotic stresses, such as drought, high salinity and cold, have a major effect on plant development and yield. An efficient response to these stresses will increase a plant's chance of survival. The levels of the plant hormone abscisic acid (ABA) rise in response to such stresses leading to the activation of signal induction pathways that induce diverse adaptive responses, such as reduction of stomata aperture, and the expression of stress-related genes [1]. Despite its reputation as a stress hormone, ABA is also involved in the control of other physiological processes. It participates in plant vegetative development in a concentration-dependent manner, stimulating growth at low concentrations and inhibiting growth at high concentrations [2], [3]. It is also essential for seed development: during maturation it promotes the acquisition of reserves and desiccation tolerance and induces dormancy by inhibiting germination [4].

ABA accumulation is dependent on the respective levels of biosynthesis and degradation. ABA synthesis in plants occurs via an indirect pathway using carotenoids, as precursors. In the final step, abscisic aldehyde is oxidised in a reaction requiring two genes, *AAO3* coding for the abscisic aldehyde oxidase and *ABA3* encoding an enzyme necessary for the sulfuration of the AAO3 molybdenum cofactor [1]. Reduction of active ABA levels is achieved by either conjugation to form a glucosyl ester or oxidation. The major oxidative pathway in Arabidopsis (*Arabidopsis thaliana*) is through hydroxylation of ABA on C-8′ by members of the cytochrome P450 monooxygenase CYP707A family [5], [6], followed by spontaneous isomerisation to phaseic acid.

Recent advances have finally enabled a complete mechanism for ABA signalling to be described (reviewed in [7], [8]) and an entire pathway from ABA perception to the induction of ABA-responsive genes has been demonstrated *in vitro* [9]. A key breakthrough has been the demonstration that the PYR (pyrabactin resistance)/PYL (PYR1-like)/RCAR (regulatory component of ABA) family of START proteins act as intracellular coreceptors of ABA by binding ABA in a gate-latch-lock mechanism that allows their interaction with the protein phosphatase 2C (PP2C) enzymes ABI1, ABI2, HAB1 and PP2CA/AHG3 [10], [11], [12]. The intracellular localisation of the START proteins means that ABA must cross the cell membrane. In its anionic form ABA is potentially membrane permeable, however, ATP-binding cassette (ABC) transporters have now been identified that transport ABA either into or out of cells [13], [14]. Putative extracellular ABA-receptors, such as GTG1 and 2, and a plastid located receptor, ABAR/CHLH, have also been identified, but remain to be linked with other ABA signalling elements [15], [16].

Water is a major input for crop production and water shortages have profound effects on yield. Understanding plant responses to water limitation is fundamental for the improvement of water use efficiency. Resistance to water deficit can occur through physiological responses that enable either drought avoidance or drought tolerance [17]. Avoidance involves the maintenance of tissue water potential despite water deficit and can be achieved by limiting water loss, for example by reducing stomata aperture and leaf surface, or improving water uptake by roots. Tolerance involves the induction of protective mechanisms, such as osmotic adjustment and synthesis of osmoprotectants, which limit cellular damage at low water potentials [18]. ABA is involved in both avoidance and tolerance mechanisms through rapid ABA-induced stomata closure and the induction of stress responsive genes.

Water stressed plants can have modified responses to pathogens and there is increasing evidence that ABA levels can impact on biotic stress responses [19]. The role of ABA seems, however, to be complex as its effect depends on the type of pathogen, its invasion strategy and the tissue affected [20]. ABA has been reported to act as a negative regulator of plant defence responses to a variety of biotrophic and necrotrophic pathogens [21] and mutants affected in ABA biosynthesis or perception have been found to be more resistant to the bacterium *Dickeya dadantii* (ex *Erwinia chrysanthemi*) [22]. Although certain ABA responses, such as stomata closure, could directly provide biotic stress resistance, defence mechanisms would also appear to be stimulated via ABA interplay with other hormones involved in pathogen resistance, such as jasmonic acid and salicylic acid [23], [24]–[26].

A limiting factor for plant amelioration is the identification of genes that can be modified to improve resistance to water deficit, while maintaining plant growth and yield when water is available. Furthermore, despite recent advances in our comprehension of ABA metabolism and signalling events, much still remains to be understood. Mutants identified in novel screens have been fundamental tools for the identification of pathway components and potential genes that can be modified to improve drought resistance. In order to identify additional loci involved in ABA accumulation or signalling, we have carried out a screen for suppressor mutants of the ABA-deficient mutant *aba3-1*. Leaf temperature, measured by thermal imaging, was used as an indication of stomata conductance under water deficit and ABA biosynthesis mutants, such as *aba3*, have colder leaves than wild-type [27]. This technique has previously been used to identify novel mutants that affect Arabidopsis stomata closure [27]–[29]. Nonetheless, the majority of mutants identified in these screens, using mutagenised wild-type populations, were affected in genes already known to be involved in ABA biosynthesis or signalling [27], [30]. Previous suppressor screens for ABA-signalling have used seed germination phenotypes [31]–[33] and to our knowledge this study is the first to use infrared thermography. Three *hot ABA-deficiency suppressor* (*has*) mutants were isolated that retained more water on drought stress and had recessive mutations that modified stomata closure in the absence of ABA deficiency. All of the mutants showed an enhanced response to ABA indicating that the three *HAS* loci encode negative regulators of ABA signal transduction. Increased susceptibility to the pathogen *Dickeya dadantii* confirmed the involvement of these loci in ABA mediated crosstalk between biotic and abiotic stress signalling pathways.

## Results

### Identification of hot *aba3-1* suppressor mutants

**Figure 1:**
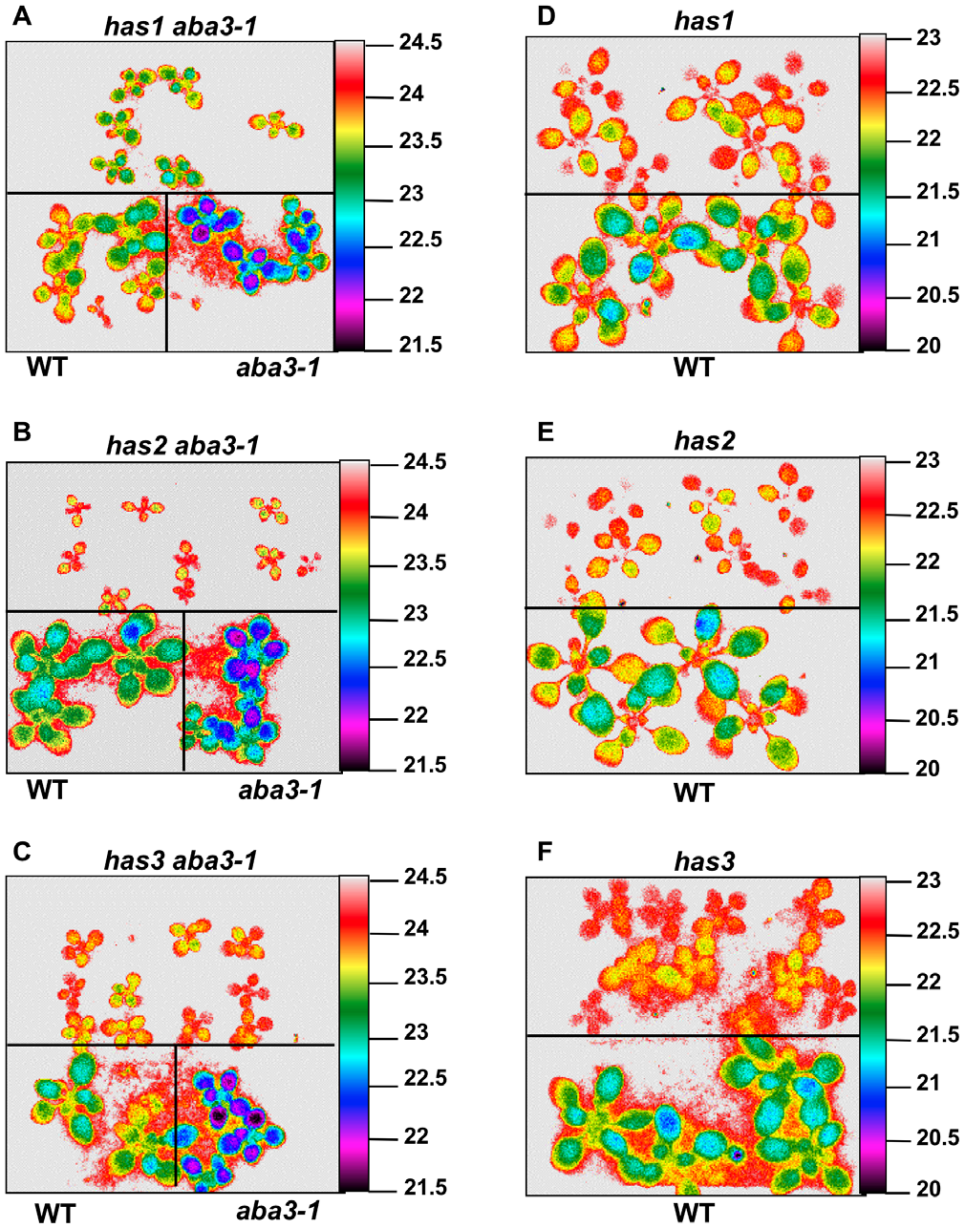
Hot leaf phenotype of *has aba3-1* and *has* mutants visualised by thermal imaging. False colour infrared image of the temperature of drought stressed plants. (A–C) leaf temperature of *has aba3-1* mutants compared to wild-type (WT) and the *aba3-1* mutant. (D–F) leaf temperature of *has* mutants compared to wild-type. Plants were 14 days old and watering had been withheld for 2 days. Scale indicates temperature (°C).

A γ- irradiation induced mutant population had been derived from seeds of the ABA deficient *aba3-1* mutant. Seedlings from M_2_ pools were screened by infrared thermography for suppression of the cold leaf phenotype observed in the original *aba3-1* mutant. Of 130 plants that exhibited a hot leaf phenotype in the primary screen, 17 showed a heritable phenotype in the M_3_ generation and were named *hot ABA deficiency suppressor (has) aba3-1* (as shown in Figure 1A-1C for *has1 aba3-1* to *has3 aba3-1*). The presence of the original *aba3-1* mutation (G3707 to A) was confirmed in the mutants by sequencing of genomic DNA. In addition, the absence of a functional sulfurylated molybdenum cofactor was verified by the absence of xanthine dehydrogenase activity, another enzyme that requires the same cofactor as AAO3 (Figure S1). This indicated that the loci were all extragenic suppressors of the *aba3-1* mutation.

**Figure 2:**
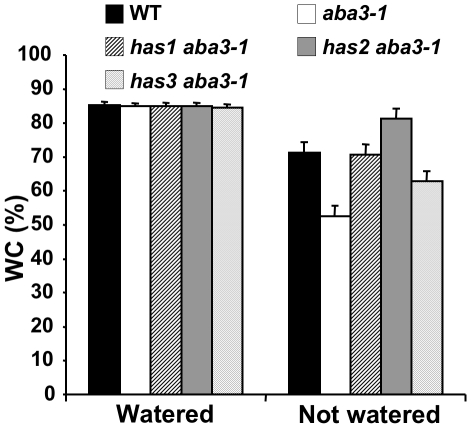
*has aba3-1* suppressor mutants show reduced water loss on progressive drought stress. Watering was withheld from three-week old plants for seven days and soil allowed to dry, not watered, or continued, water control. Water content (WC) was calculated as the % (w/w) of rosette weight corresponding to water, as determined from plant weight before and after freeze-drying. WT, wild-type. Error bars represent SE values (n = 4). Similar results were obtained in 2 independent experiments.

The *aba3-1* mutant cold leaf phenotype is associated with increased transpiration and mutant plants are less resistant to water deficit than wild-type [34]. To confirm that the hot leaf suppressor mutations were related to improved resistance to water stress, 3-week old plants were examined for their water loss when subjected to a progressive drought stress. Water was withheld for 7 days and soil allowed to dry so that all plants were subjected to a similar soil water deficit. Seven of the suppressor mutants clearly retained more water on water deficit than the mutant *aba3-1* alone (as shown in Figure 2 for *has1 aba3-1* to *has3 aba3-1*). These mutants were backcrossed to the *aba3-1* mutant and the segregation of the suppressor phenotype analysed. Among the suppressor mutations, *has1*, *2* and *3* were chosen for further analysis because they segregated as single Mendelian recessive loci (Table 1), exhibited clear hot leaf phenotypes (Figure 1 A–1C) and had a similar plant stature to that of the original *aba3* mutant in non-stressed conditions.

**Table 1:** Segregation analysis of three *HAS* loci in backcrosses to original *aba3-1* mutant indicates that the three mutations are recessive.

Cross^a^	F_1_ progeny IR phenotype	F_2_ progeny IR phenotype	
		cold∶hot	chi-square test
*aba3-1*×*has1 aba3-1*	cold	150∶54	0.24, p>60%^b^
*aba3-1*×*has2 aba3-1*	cold	61∶29	2.50, p>10%^b^
*aba3-1*×*has3 aba3-1*	cold	82∶33	0.84, p>30%^b^

Infrared (IR) thermography was carried out on 14-day-old plants that had been subjected to water deficit for 2 days.

a All crosses are written as female parent×male parent.

b Null hypothesis of 3∶1 *HAS* (cold)∶*has* (hot); degrees of freedom = 1. p, probability.

The three *has aba3-1* mutants were identified from different M_1_ seed pools indicating that they were generated from different mutation events. For mapping of these defective loci, crosses were carried out between *has1 aba3-1*, *has2 aba3-1* and *has3 aba3-1* in the Columbia (Col-0) accession and the *aba3-11* mutant in the Landsberg *erecta* (L*er*) accession. The *aba3-11* mutant, previously named VI-48, was identified in an earlier thermal imaging screen [27]. As the mutant lacked xanthine dehydrogenase activity, it was suggested to be an *aba3* allele. Sequencing of the *ABA3* gene identified a point mutation in *aba3-11* (G2085 to A) that would result in an amino acid change Gly-280 into Ser.

Linkage analyses to molecular markers enabled the localization of the *HAS* loci to three different chromosomal positions (Table 2). The presence of the *CYP707A1* gene, involved in ABA catabolism, in the mapping interval for the *has3* mutation made it a strong candidate for being the affected locus. Nevertheless, *has3* is not a *cyp707a1* mutant allele as allelism tests carried out with *cyp707a1-1* resulted in phenotypic complementation (Figure S2). Furthermore, no mutation was identified in the *CYP707A1* DNA sequence in the *has3* mutant (from 1549 bp upstream of the ATG to 326 bp downstream of the STOP).

**Table 2:** Chromosome location of three *HAS* loci.

Locus	Chromosome	Location in Mb (AGI)	BAC or YAC interval
*HAS1*	3	3.157–3.651	F14P13 – F24K9
*HAS2*	4	8.255–9.362	FCA1 – FCA6
*HAS3*	4	10.366–10.609	F13C5 – F24J7

Mapping was carried out with PCR-based SSLP markers. The BAC or YAC interval indicates the BACs or YACs containing the markers delimiting the mapping interval.

The mapping interval containing the *HAS1* locus included the *PP2CA/AHG3* gene whose mutation results in ABA-hypersensitivity. Nevertheless, *pp2ca/ahg3* mutant phenotypes are very different to those of *has1*, although mutants have ABA-hypersensitive stomata closure, they also show ABA-hypersensitive root growth and seed germination, with the latter being particularly marked [35], [36]. In addition, the *pp2ca/ahg3* single mutant is not resistant to water deficit on rapid dehydration and it is only in combination with other PP2C mutants that enhanced resistance to drought stress is observed [36]. It seems unlikely, therefore, that *has1* is an allele of *pp2ca/ahg3*.

### Hot *has* phenotypes are not due to increased ABA levels

To investigate whether the *has* mutants had phenotypes in the absence of the *aba3-1* mutation, crosses were carried out with wild-type Col-0 to separate the mutations. Each *has aba3-1* mutant was crossed to the Col-0 wild-type (Figure S3; Protocol S1). F_2_ progeny that were heterozygous for *aba3-1* were selected using a dCAPS marker for the *aba3-1* mutation. The infrared phenotype of their F_3_ descendants was then examined and F_2_ parents with no cold offspring were retained, as the cold leaf phenotype in the quarter of the plants homozygous for the *aba3-1* mutation would only be suppressed in the presence of a homozygous *has* mutation. The dCAPS marker was then used to identify F_3_ plants that were also homozygous for the wild-type *ABA3* allele, and these mutants were then named *has*.

**Figure 3:**
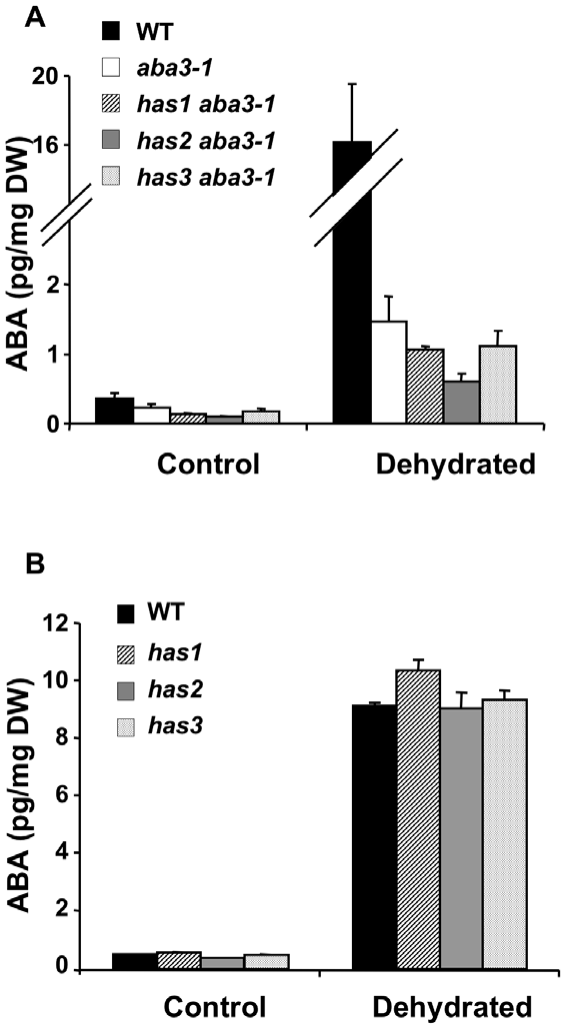
The *has* loci do not modify ABA accumulation. A, The ABA-deficient phenotype is not suppressed in *has aba3-1* mutants. Results presented are representative of those obtained in 2 independent experiments. B, *has* mutants do not have higher ABA levels than wild-type. ABA contents were determined in rosette leaves after 4 h of dehydration (dehydrated) and compared with non-dehydrated (control) plants. No significant difference was found in Student *t*-tests comparing *has aba3-1* to *aba3-1* or *has* to wild-type. WT, wild-type; DW, dry weight. Error bars represent SE values (n = 3).

Analysis of *has* plants by infrared thermography showed that all three *has* mutants had a higher leaf temperature when compared to the wild-type (Figure 1D–1F). Prior to or after rapid dehydration, no difference in ABA accumulation was detectable in *has* mutants compared to wild-type and in *has aba3-1* mutants compared to *aba3-1* (Figure 3). Therefore it is unlikely that altered ABA metabolism causes the hot phenotype conferred by *has* mutations.

### Effect of *has* mutations on vegetative development and photosynthesis

**Figure 4:**
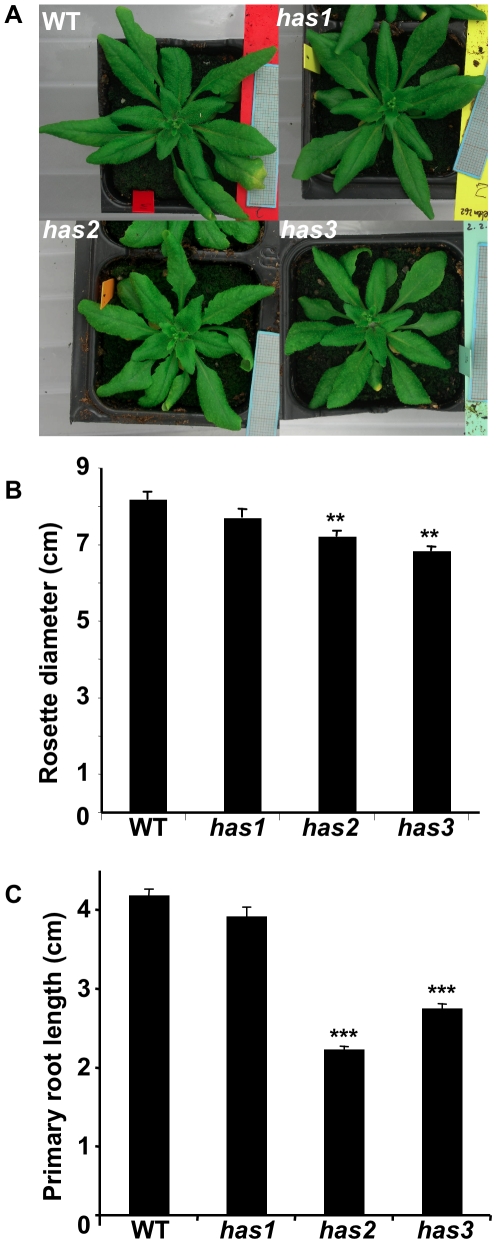
Reduced rosette size and root length of *has2* and *has3* mutants. Wild-type *has1*, *has2* and *has3* A, rosettes grown in long-day photoperiod for 14–18 days. B, rosettes grown in short-day photoperiod for 55 to 73 days. C, primary root length of 13 d old seedlings grown in long-day photoperiod. WT, wild-type. Error bars represent SE values (b, n = 4; c, n = 24). Similar results were obtained in 2 independent experiments. Student *t*-test p<1%, ** or p<0.1%, ***.

When plants bolted, the rosettes of *has2* and *has3* mutants grown in long-day photoperiods were slightly smaller than those of wild-type (Figure 4A) although at maturity these differences in rosette size were less evident. This size reduction was also observed for plants grown in a short-day photoperiod (Figure 4B) and for 14 d-old drought-stressed plants (Figure 1 B, C, E and F). Furthermore, *has2* and *has3* had a reduced number of rosette leaves at flowering (wild-type: 20±0.7; *has1*: 19.3±0.9; *has2*: 16.3±0.7; *has3*: 16.75±1.0). Flowering time was, however, not affected in *has1* and *has2* in long- and short-day conditions or for *has3* in long-day conditions. Interestingly, in short-day conditions, bolting of floral stems was earlier in the *has3* mutant (wild-type: 62.3 days±0.9; *has3*: 55.7±0.7), indicating that in certain circumstances *has3* is an early-flowering mutant. Primary root length was also reduced in the *has2* and *has3* mutants, in accordance with the reduced rosette size (Figure 4C).

As increased leaf temperatures and modified growth characteristics could result from defects in the transfer of light energy during photosynthesis, assays of photosynthetic capacity were carried out. The level of photosynthesis was measured using net CO_2_ uptake at different CO_2_ concentrations in an oxygen atmosphere of 21% (v/v) (ambient air) or 0.5% (v/v) (non-photorespiratory conditions). All three *has* mutants showed wild-type CO_2_ responses indicating that photosynthesis is not affected in the mutants (Figure S4; Protocol S2). Furthermore, no significant modifications of stomatal density (abaxial or adaxial leaf surface) were observed compared to the wild-type (Figure S5; Protocol S3). This is consistent with the fact that leaf temperature differences were only observed under conditions of water deficit.

### Resistance to rapid dehydration and expression of stress responsive genes

**Figure 5:**
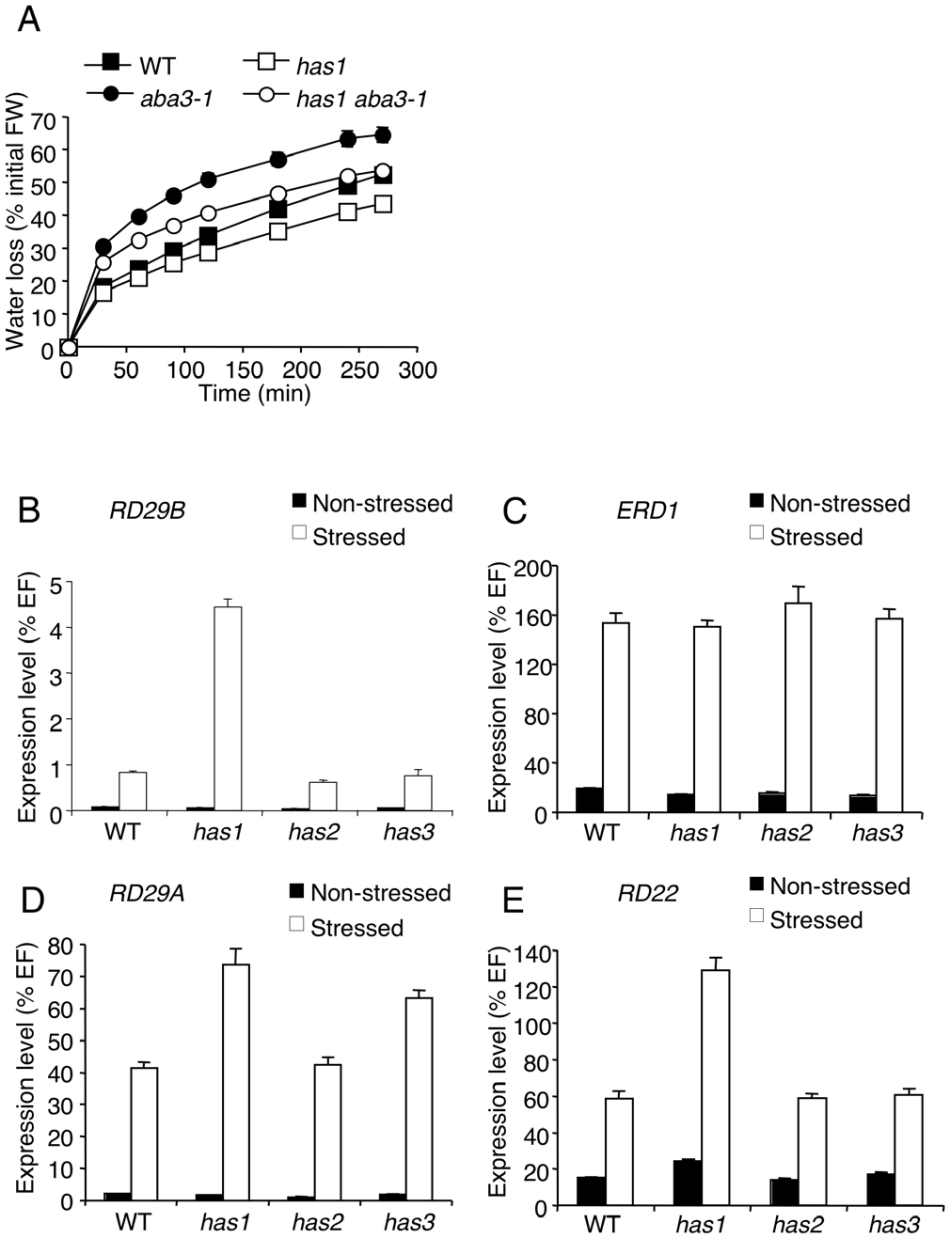
The *has1* mutation improves resistance to rapid dehydration and causes the overexpression of *RD29B*, *RD29A* and *RD22*. A, Rapid dehydration of plants carrying the *has1* mutation alone or with the *aba3-1* mutation. Water loss is expressed as a percentage of the initial fresh weight (FW). Error bars represent SE values (n = 4). Results presented are representative of those obtained in 4 independent experiments. Quantitative RT-PCR analysis of drought inducible gene expression in wild-type and *has* mutants, B, *RD29B*, C, *ERD1*, D, *RD29A* and E, *RD22* transcript abundance in leaves after 4 h at a water-deficit equivalent to the loss of 25% (w/w) of the fresh weight (stressed) compared to controls (non-stressed). Steady-state mRNA levels are represented as a percentage of the constitutive *EF1α-4a* gene (EF) abundance. Error bars represent SE values (n = 3). WT, wild-type. Similar results were obtained for samples derived from 2 independent plants.

As well as being less drought-resistant, the *aba3-1* mutant shows increased water loss in rapid dehydration assays of detached rosettes [34]. Similar assays were performed to determine whether the suppressor loci also improved resistance to rapid dehydration. The only mutation that significantly reduced water loss when subjected to this rapid and drastic water deficit was *has1* either alone or in the *aba3-1* mutant context (Figure 5A).

To assess the responses of *has* mutants to water deficit at the molecular level, we examined the expression of four drought-inducible genes, known to differ in the *cis*-acting elements present in their promoters. Expression of the *responsive to dehydration (RD)29B* gene is mainly controlled via ABA and in accordance its promoter contains two ABRE [37], [38]. The *RD22* promoter contains binding regions for MYC and MYB transcription factors, involved in the ABA-dependent induction of genes during late drought responses [39], [40]. *RD29A* expression is controlled by both ABA-dependent and independent pathways [41] and as well as an ABRE its promoter contains a dehydration responsive element (DRE), that is recognised by a subfamily of transcription factors with an APETALA2 domain and is required for ABA-independent drought-induced gene expression [42]. The *EARLY RESPONSIVE TO DEHYRATION* (*ERD)1* gene is also induced via ABA-independent pathways, but that do not involve DRE *cis*-elements; two distinct promoter sequences bind either NAC (NAM, ATAF, CUC2) family or ZFHD (zinc-finger homeodomain) transcription factors [43]–[45].

In agreement with previous studies, all four genes were induced by water deficit in the wild-type (Figure 5B–5E). Expression levels of *ERD1* transcripts were similar to those of wild-type for all three mutants indicating that the *HAS* loci are not involved in the ABA-independent signalling pathway that induces this gene (Figure 5C). Furthermore, in the *has2* mutant *RD29B*, *RD22* and *RD29A* transcript abundance were also equivalent to wild-type gene (Figure 5 B, D and E). In contrast, these three genes were overexpressed in *has1*, suggesting that the *HAS1* locus is involved in both ABA-dependent and independent signalling pathways. The *has3* mutant appears to be affected in an ABA-independent pathway that is separate from that inducing *ERD1*, as only *RD29A* transcript abundance was significantly increased (Figure 5D).

### *has* mutants show ABA-hypersensitive stomatal closure

**Figure 6:**
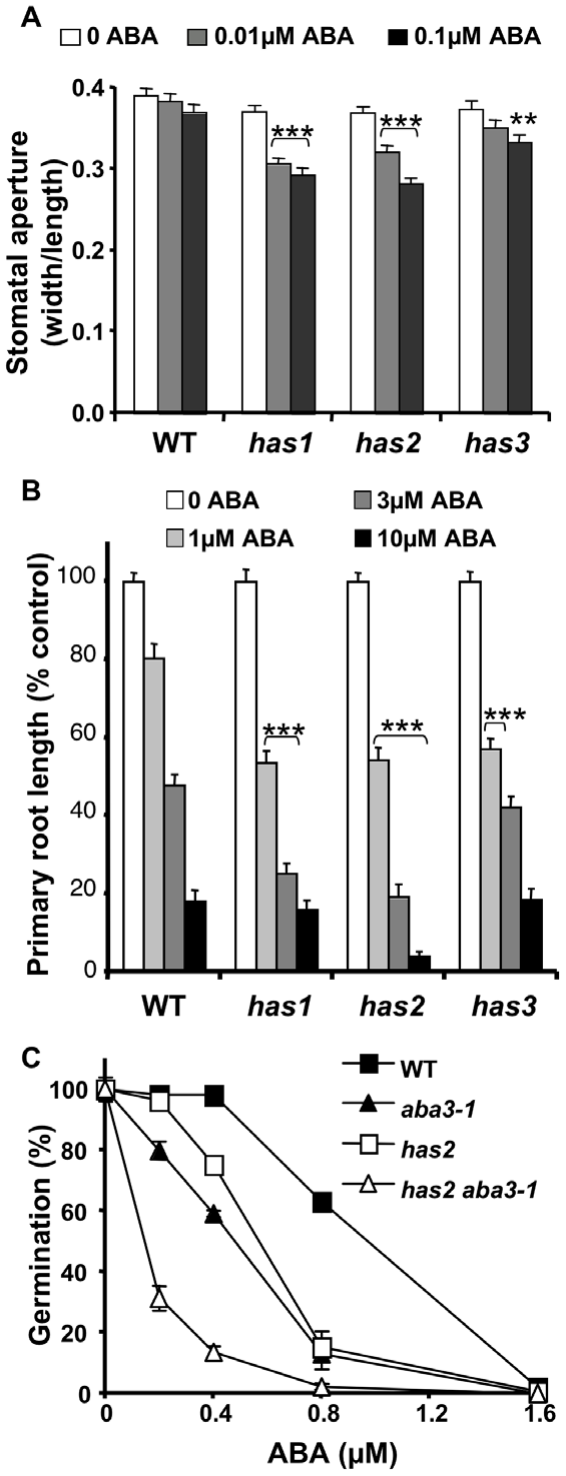
*has* mutants show ABA-hypersensitivity compared to wild-type. A, Induction of stomatal closure by ABA in wild-type and *has* mutants. Stomata aperture ratios (width/length) were measured after a 2 h pre-treatment in the light in stomata opening solution followed by a 3 h incubation with or without ABA at the concentrations indicated. Data are means ± SE of 3 independent experiments with 40 apertures measured per experiment and condition. Significance in Student *t*-tests comparing samples with and without ABA for the same genotype; **, p<1% or ***, p<0.1% level. B, ABA inhibition of root growth. Primary root length of 13 d old seedlings grown in long day photoperiod ABA was included in the growth media at the concentrations indicated. Error bars represent SE values (n = 24). Results presented are representative of those obtained in 2 independent experiments. C, ABA inhibition of seed germination after 7 d at 25°C. ABA was included in the growth media at the concentrations indicated. Germination was determined from the number of seedlings with green cotyledons compared to the total number of seeds sown. Error bars represent SE values (n = 3). Results presented are representative of those obtained in 2 independent experiments. Significance in Student *t*-test when comparing mutant and wild-type at a given ABA concentration, p<0.1%, ***. WT, wild-type.

As the increased leaf temperature in the *has* mutants did not result from either increased ABA accumulation, reduced stomata density or reduced dissipation of heat due to altered photosynthetic capacity, the response of stomata to ABA was examined. After 5 h in the light in stomata opening solution *has* mutant and wild-type stomata opened to the same extent (Figure 6A; Figure S6). Low ABA concentrations resulted in a limited reduction in stomata aperture for wild-type that was not significantly different from untreated stomata (Figure 6A). In contrast, all three mutants showed a significant reduction in stomata aperture in the presence of ABA, with *has1* and *has2* being more ABA-hypersensitive. At high ABA concentrations wild-type showed the expected reduction in stomatal aperture, but closure was no longer significantly different in *has* mutants (Figure S6). An increased response of stomata to low ABA concentrations would be expected to result in elevated leaf temperatures and improved drought resistance on water deficit.

### Root growth and germination responses to ABA

Analyses of root growth and germination were carried out to determine whether *has* mutant ABA-hypersensitivity extends to other tissues than guard cells. ABA inhibition of root growth and germination is dose dependent as can be seen by a reduction of primary root length and the number of germinating seeds for wild-type (Figure 6B–6C). Root growth of all three *has* mutants was significantly reduced on 1 µM ABA compared to wild-type (Figure 6B). At higher ABA concentrations *has2* root growth was still hypersensitive, whereas *has1* roots were only more sensitive at 3 µM, and the *has3* mutant was inhibited in a similar manner to wild-type. In contrast, ABA-hypersensitive germination was only clearly observed for *has2* seeds (Figure 6C). The *has2* mutant seeds also showed increased dormancy and sensitivity to the gibberellin biosynthesis inhibitor paclobutrazol, in accordance with the ABA-hypersensitive phenotype (Figure S7).

### Biotic stress response

**Figure 7:**
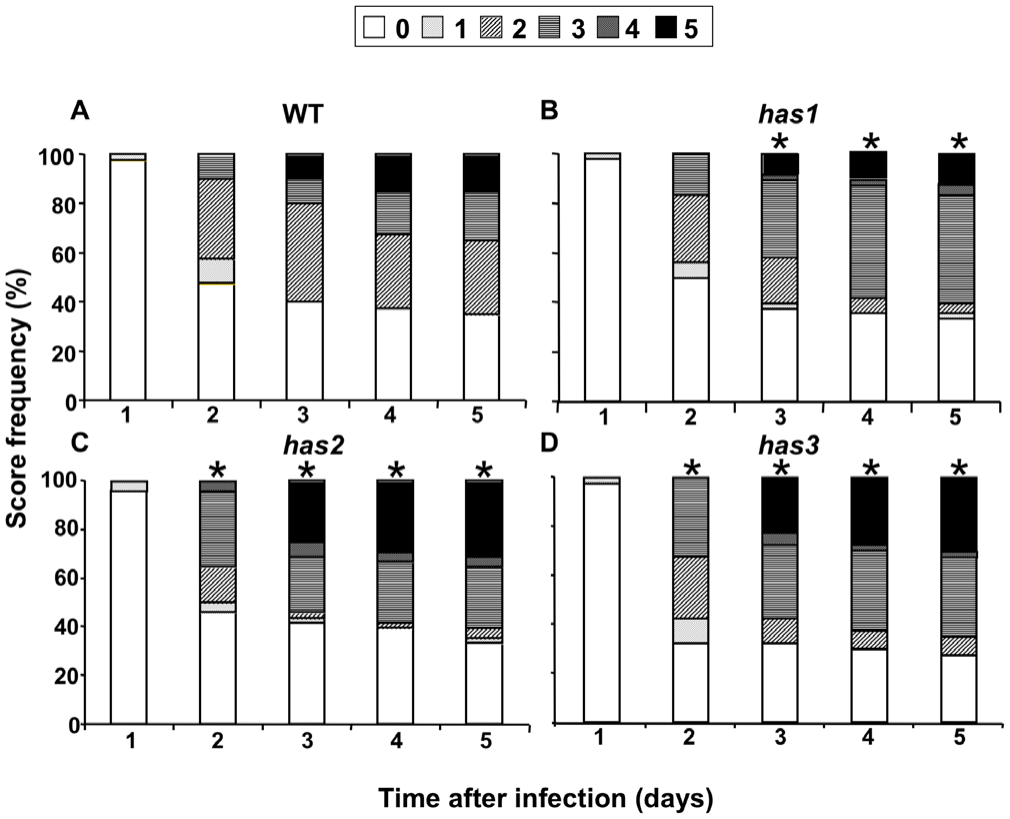
Level of *Dickeya dadantii* bacterial infection of *has* mutants. Degree of infection of wild-type, *has1*, *has2* and *has3* plants (40 to 48 individuals) and its evolution over 5 days after inoculation. The scale of infection is denoted by: 0: no symptom; 1: maceration limited to the inoculation point; 2: maceration extends from the infection point; 3: maceration covers half the lamina; 4: maceration spread over the whole lamina; 5: maceration spread over the whole leaf (lamina and petiole). Similar results were obtained in 2 independent experiments. *, significant differences, p<0.05%, between wild-type and mutants in Fisher tests comparing infection scores of ≥3.

Recent results have emphasized the involvement of ABA in plant responses to a large number of pathogens [46], [47]. As the genes affected in the *has* mutants are potential targets for the improvement of drought resistance it was important to verify whether their mutation modifies disease resistance. In order to address this question, the pectinolytic bacterium *Dickeya dadantii* was used in infection assays. ABA biosynthesis and signalling mutants exhibit improved resistance to this pathogen [22], [48], which causes soft rot of leaves, stems and storage organs. The disease progression was evaluated by the level of maceration observed between one and five days after inoculation (Figure S8). All three *has* mutants were less resistant to *Dickeya dadantii* than wild-type (Figure 7). The degree of sensitivity varied between the mutants, with *has1* being the least affected and *has2* and *has3* clearly more susceptible than the wild-type.

## Discussion

This paper describes the isolation of three new mutants affected in stomata responses to ABA using a novel screen for suppression of the cold leaf phenotype exhibited by the ABA-deficient mutant *aba3-1*. No allele was identified twice independently indicating that the screen carried out was not at saturation. It should be possible, therefore, to extend this screen in the future and identify further new mutants. Leaf temperature could be altered by other physiological parameters than stomata aperture, such as the amount of epicuticular wax [49], and less than half of the suppressor mutants initially identified retained more water on progressive drought stress. This demonstrates that although infrared thermography is an excellent tool for high-throughput screening and has the advantage of being non-destructive, additional criteria are required to distinguish mutants affected in stomatal conductance.

The *aba3-1* mutant has reduced levels of ABA in vegetative tissues under stressed and non-stressed conditions, nevertheless residual ABA levels are higher than in most other ABA-deficient mutants [34]. Suppression of an ABA-deficient phenotype by restoring ABA content would theoretically be more easily detected using such a mutant. No suppressor mutant was identified, however, where ABA levels had been re-established in vegetative tissues (Figure 3). Increased ABA content could be achieved by an augmentation of the expression of an ABA biosynthesis gene or by reducing ABA catabolism, for example through the mutation of a *cis*-acting regulator. The *NCED* and *CYP707A* multigene families encode enzymes that carry out key steps of ABA biosynthesis or catabolism, respectively [50], [51]. The desulfo-molybdenum cofactor produced by ABA3 is required for the catalysis of the last step of the biosynthesis pathway, and acts downstream of NCED activity. Increasing precursors prior to this last step might not, therefore, significantly increase ABA production; the *aba3* mutation is likely to cause a bottleneck that would already be saturated by precursors. Reduction of ABA catabolism should, however, be independent of such an effect.

Of the four *CYP707A* genes implicated in the inactivation of ABA, two have been demonstrated to contribute to the regulation of transpiration, *CYP707A1* and *CYP707A3* [51], [52]. Reporter gene analysis indicates that they are mainly expressed in stomata and vascular tissue, respectively and the *cyp707a1* mutant showed ABA-hypersensitive stomata closure and germination [52], [53]. In high humidity conditions ABA levels were nearly three-fold higher in a *cyp707a3* mutant than those of wild-type controls, whereas a *cyp707a1* mutant showed only a limited ABA increase, despite both mutants exhibiting similar reductions in stomata aperture in these conditions [52]. Consequently, modifications in ABA levels might not be detected even if *CYP707A1* expression is affected. Although this gene is present in the mapping interval for the *has3* mutation allelism tests and sequencing confirmed that the *has3* is not a *cyp707a1* mutant allele.

### Three independent mutations generating moderate ABA hypersensitive phenotypes

**Figure 8:**
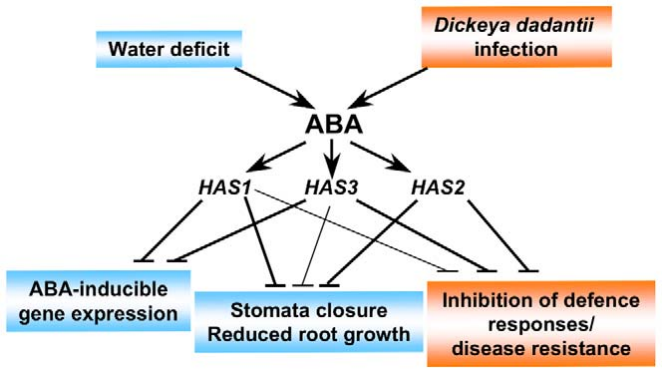
Schematic representation of the *HAS* loci mode of action as putative negative regulators in ABA signalling responses to *Dickeya dadantii* infection and water deficit. Responses to water deficit and *Dickeya dadantii* infection are highlighted in blue or orange, respectively. Line width reflects extent of locus effect on a given response.

The three *has* mutants are affected at independent loci (Table 2), and their phenotypic differences, in particular the downstream target genes induced on dehydration (Figure 5B–5E), indicate that they are involved in different ABA response pathways (Figure 8). As the mutations are inherited as recessive loci and cause ABA-hypersensitivity the three *HAS* genes are predicted to encode negative regulators of ABA signalling. Not all ABA-hypersensitive mutants are drought-resistant, such as *fry1* and *sad1*, despite inducing the expression of ABA response genes [54], [55]. All three *has* mutants, however, retained more water on progressive drought stress and showed ABA-hypersensitivity in both stomata closure and root growth (Figure 2; Figure 6A–6B). Interestingly, the *has* mutant hypersensitive phenotypes in vegetative tissues were visible at relatively low ABA concentrations (Figure 6) yet had little or no effect on vegetative growth or photosynthesis under well-watered conditions (Figure 4; Figure S4). This indicates that *has* mutant responses are sufficient to reduce water loss when soil water potential is low, while minimizing the effect on photosynthesis and growth when water is available. On water deficit, however, *has* mutants were smaller than wild-type which could be due to direct ABA effects on growth due to hypersensitivity or as a consequence of increased stomata closure reducing photosynthesis. Functional redundancy between members of multigene families encoding elements of the ABA-signalling pathway can restrict mutant phenotypes. Mutants in PP2C family members have enhanced or constitutive ABA-response phenotypes when combined together [36]. Similar functional redundancy could explain the limited growth defects of *has* mutants.

### *has3* exhibits mild ABA-hypersensitive phenotypes

Of the three mutants, *has3* showed the weakest ABA-hypersensitive responses; in both leaves and roots significant hypersensitivity was only observed at one ABA concentration (Figure 6A–6B). Yet this mutant had the most clear developmental phenotypes, including early flowering in short day conditions and reduced rosette size and root length (Figure 4). Furthermore, of the four drought-inducible genes examined only *RD29A* expression was more strongly increased by dehydration in the mutant compared to wild-type (Figure 5D). This gene contains both ABRE and DRE *cis*-elements in its promoter, and the *RD29B* gene with ABRE was not induced, suggesting that induction is ABA-independent. This implies that although *HAS3* affects ABA responses and stomata aperture, it also induces drought avoidance mechanisms that respond to water-deficit by modifying vegetative growth, and that *HAS3* acts through both ABA-dependent and independent signalling pathways.

### The *has2* mutation causes ABA hypersensitivity in stomata, roots and seeds

Like many previously identified recessive, drought-resistant mutants, *has2* ABA hypersensitivity was not limited to stomata and roots, but also affected ABA responses in seeds (Figure 6C). In agreement with the role for ABA in seed dormancy imposition, the increased ABA sensitivity of *has2* seed correlated with delayed germination and an increased requirement for GAs biosynthesis (Figure S7). As *has2* ABA responses were affected in all tissues examined this suggests that *HAS2* function is involved in early or common ABA signalling events. Expression in *has2* of the four drought-inducible genes examined was equivalent to that of wild-type (Figure 5B–5E). As the transcription of these genes is induced on drought via diverse regulatory networks this suggests that the modification of ABA responses in *has2* is either through the induction of genes in an as yet uncharacterised signalling network or does not involve transcriptional activation. The mapping interval established for the *has2* mutant does not contain any previously characterised gene whose mutation causes drought resistance and ABA hypersensitivity, the gene affected is likely, therefore, to encode a novel negative regulator of a primary ABA signalling pathway.

### The *HAS1* locus is an excellent candidate for improving plant drought resistance

The phenotypes observed in the *has1* mutant were relatively limited, but very marked, with the only differences to wild-type being observed in response to stress or ABA; reduced water loss on rapid dehydration and progressive drought, enhanced responses of stomata and roots to ABA and increased induction of ABA response genes on water deficit. These data indicate that the *HAS1* gene is specifically required for ABA responses that improve drought resistance through rapid response mechanisms (Figure 8). As mentioned above, mutants with increased stomata closure are not always resistant to water-deficit. However, to our knowledge all of the mutants characterised to date as having ABA-hypersensitive stomata closure and root elongation also exhibit ABA-hypersensitive seed germination making *has1* a new category of mutant.

### Overlap between ABA induced responses to *Dickeya dadantii* and drought stress occurs at multiple levels

Recent evidence has implicated ABA biosynthesis and signalling pathways in crosstalk between responses to both abiotic and biotic stress [48]; mutants can be either more susceptible or resistant depending on the pathogen [20]. This means that for genes involved in ABA responses to be exploited in improving drought resistance their effect on biotic stress responses should be limited. ABA-deficient mutants were more resistant to infection by the pectinolytic bacteria *Dickeya dadantii* [22] whereas the three *has* loci were more susceptible (Figure 7). As all three mutants show enhanced ABA responses this confirms that increasing ABA signalling exacerbates *Dickeya dadantii* susceptibility. Furthermore, the *HAS* genes appear to be involved in diverse aspects of ABA-signalling, yet for each the ABA hypersensitivity induced by their mutation increased *Dickeya dadantii* susceptibility (Figure 6; Figure 7). Nevertheless, disease symptoms for *has1* were weaker than for *has2*, although both mutants exhibited similar levels of ABA hypersensitivity in vegetative tissues. This demonstrates that crosstalk between ABA induction of responses to *Dickeya dadantii* and drought stress occurs at more than one point in ABA signalling responses (Figure 8). Modulation of plant biotic stress responses by ABA is dependent on the pathogen concerned and it will be interesting to examine the susceptibility of the *has* mutants to other phytopathogens. Depending on the identity of the *HAS1* gene it could be a suitable target for amelioration of plant drought resistance as its mutation reduced water loss, yet had only limited effects on growth and *Dickeya dadantii* susceptibility.

In conclusion, the novel suppressor screen described in this paper has identified three new mutants that are hypersensitive to relatively low ABA levels and yet display little or no growth defects in well-watered conditions. Furthermore, biotic stress resistance for one mutant is only slightly compromised. The future identification of the *HAS* genes should yield new components of pathways involved in both abiotic and biotic stress resistance.

## Materials and Methods

### Plant material

The *aba3-1* mutant, Col-0 accession, was provided by M. Koornneef [34]. The *aba3-11* mutant, previously termed VI-48, is in the L*er* accession [27]. Dry *aba3-1* seeds were irradiated in a 1.5 ml microtube at room temperature with γ-rays generated by a Co^60^ source (CIGAL, Cisbio International, http://www.cisbiointernational.fr/) with a dose of 300 Gy. One hundred M_2_ seed pools were then produced, each derived from 25 M_1_ plants. For the 17 suppressor mutants showing a heritable hot leaf phenotype, M_4_ seed lots were generated from 8 individual plants and analysed by thermal imaging in order to identify a homozygous lot that presented a homogenous phenotype. Absence of xanthine dehydrogenase activity was determined using native gel electrophoresis as previously described [56]. Two successive backcrosses were performed with the original *aba3-1* mutant and phenotypes confirmed in each generation.

### Infrared thermography

For screening of the γ-ray mutagenised *aba3-1* population 50–60 M_2_ seeds, mixed with 1.5 ml of Fontainebleau sand, were sown onto the surface of 9 cm×9 cm pots. The pots contained a 50∶50 (v/v) mixture of compost and sand and were prepared and cultured as described by Merlot et al. [27]. More than 400 M_2_ seeds were screened from each of the 100 pools. For confirmation of phenotypes, surface-sterilised seeds from individual plants were grown initially *in vitro* on Arabidopsis Gamborg B5 media (Duchefa; http://www.duchefa.com/) supplemented with 30 mM sucrose, with 3 days at 4°C in the dark followed by 3 days at a light intensity of 50 µmol m^−2^ sec^−1^ with a 16 h photoperiod at 20°C. Seedlings were then transplanted to 9 cm×9 cm pots covered with a layer of Fontainebleau sand and cultured as above. Thermal imaging was carried out as previously described [27] using a Thermacam PM250 infrared camera (Inframetrics, FLIR Systems; http://www.flir.com) equipped with a 16° lens.

### Water loss assays

Rapid dehydration assays were carried out using 3-week-old plants grown in compost (Tref Substrates, http://www.trefgroup.com/) in the glasshouse (18–28°C, minimum 13 h photoperiod). Four rosettes per genotype were cut from the root system and water loss was measured as previously described [57]. Resistance to progressive drought stress was carried out in the same 50∶50 (v/v) mixture of compost and sand used for thermal imaging in 67 cm^3^ baskets with a 0.5 cm layer of vermiculite at the bottom; pots were adjusted to contain the same weight of growth medium. Eight plants per genotype were transplanted and grown in the glasshouse for three weeks. For each genotype watering was then stopped for four plants. At the start of the experiment each plant/pot was weighed and the rate of water loss from soil followed by weighing pots each successive day at the same hour. The relative positions of pots were modified after each weighing to avoid position effects on drying. Water availability to plants from soil was calculated each day and confirmed to be similar (maximum variance 10% (w/w)). After seven days, soil was essentially dry (<0.005 g water/g compost-sand) and *aba3-1* plants had reached wilting point. Rosettes were harvested, weighed, freeze-dried and reweighed. Plant water content was calculated from the difference in rosette weight before and after freeze-drying.

### DNA sequencing and genetic analyses

Genomic DNA was extracted from flower buds as described by Doyle and Doyle [58] and sequencing of the *ABA3* gene performed using the Applied Biosystems DNA sequencing kit (BIGDYE TERMINATOR, version 3.0) and the ABI Prism 310 genetic analyzer (Applied Biosystems; http://www.appliedbiosystems.com). For mapping, crosses were performed between *aba3-11* and *has1* to *3 aba3-1*. F_2_ progeny with hot leaf phenotypes were selected by thermal imaging and DNA extracted in a 96-tube format either as described by Macquet et al. [59] or Simon et al. [60]; 147, 157 and 193 F_2_ plants were selected for *has1*, *has2* and *has3*, respectively. The different progeny were genotyped using simple sequence length polymorphism (SSLP) markers and approximate genome positions determined based on recombination percentages. The mapping interval was then reduced using additional recombinants selected by genotyping series of plants with markers at each extremity of the interval (Table S1), followed by determination of their phenotype by infrared thermography. In total mapping populations of 1600, 570 or 2000 F_2_ individuals were analysed for *has1*, *has2* and *has3*, respectively.

### ABA content determination

Rosettes submitted to rapid dehydration or from control plants were frozen in liquid nitrogen and freeze-dried. Individual measurements were obtained from the leaf tissue in a single rosette. Dried rosettes were ground in 3 ml of extraction solvent (acetone, water, acetic acid, 80/19/1, v/v/v), in which 50 ng of ^2^H-ABA ((-)-5, 8′, 8′, 8′-d4 ABA purchased from Irina Zaharia, Plant Biotechnology Institute, National Research Council Canada, http://www.nrc-cnrc.gc.ca) was added as an internal standard. Samples were centrifuged and the supernatant recovered, the pellet was then resuspended in a further 2 ml of extraction solvent by sonication, recentrifuged and the supernatants combined. The extraction solvent was then evaporated and the residue resuspended by sonication in 0.5 ml of chromatography mobile phase (acetonitrile, water and acetic acid, 50/50/0.05 v/v/v) and filtered through a 1.6 µm GFA filter (Whatman, http://www.whatman.com/). ABA was quantified using a LC-ESI-MS-MS system (Quattro LC, Waters, http://www.waters.com) in positive ionisation and multiple reaction monitoring mode.

### Allometry measurements

After 1 week of *in vitro* culture (16 h photoperiod, 50 µmol m^−2^ sec^−1^, 20°C, 70% relative humidity), plants were transplanted to soil in growth chambers (21°C day, 17°C night, 150 µmol m^−2^ s^−1^ light intensity, short days 8 h photoperiod or long days 16 h photoperiod, 65% relative humidity). Rosette diameter was determined from photographs using ImageJ 1.34S software (Freeware, National Institute of Health, USA, http://rsb.info./nih.gov/ij/). Flowering time was taken as the day when the first flowering stem measured 0.5–1 cm and the number of rosette leaves counted.

For root length measurements, surface sterilised seeds were sown *in vitro* on Arabidopsis Gamborg B5, 30 mM sucrose media for 2 d as described above. Germinated seeds with protruding radicles were then transferred to 144 cm^2^ square plates containing the culture media described by Estelle and Somerville [61] with 1% (w/v) sucrose and 1.2% (w/v) Phytablend agarose (Caisson Laboratories, http://www.caissonlabs.com/) which were then incubated horizontally for 1 d and then vertically for a further 11 d in the same conditions. The plates were scanned and the length of the primary root determined using ImageJ 1.34S.

### Stomatal aperture and root length responses to ABA

Assays of ABA induced stomatal closure were performed essentially as described by Pei et al. [62]. Plants, grown for 5- to 6- weeks in soil in a growth chamber (21°C day, 17°C night, 65% RH, 160 µmol m^−2^ s^−1^ light intensity, 16 h photoperiod), were watered and kept at 95% RH overnight before leaf harvest. Rosette leaves were detached from plants and floated abaxial side up on stomata opening solution (20 mM KCl, 10 µM CaCl_2_, 5 mM MES-KOH, pH 6.15). Leaves were incubated for 2 h in a growth cabinet (23°C, light intensity 150 µmol m^−2^ s^−1^) then ABA (JunDa Pharm Chem Plant Co. LTD.; http://jundapharmchem.en.ecplaza.net/) was added to 0.1 and 0.01 µM and incubation continued for a further 3 h. ImageJ 1.34S was used to measure stomatal apertures on light microscopy images of epidermal peels, obtained from leaves adhered to double-sided sticky tape on a microscope slide and scraped with a scalpel to remove the bulk of the leaf tissue [63]. Acquisition of a ll epidermal images and measurements of stomata aperture were performed without knowledge of the genotype being examined.

For root responses to ABA, plants were grown on vertical plates as described above for biometry measurements, except that ABA was added to the media to a final concentration of 1 µM, 3 µM or 10 µM. Measurements of root length were performed with ImageJ 1.34S as described above.

### Germination experiments

For dormancy assays, surface-sterilised dry seeds were sown in triplicate in Petri dishes containing 0.5% (w/v) agarose and placed in a growth chamber (16 h photoperiod, 25°C, 70% relative humidity). Germination was scored each day based on radicle protrusion. For paclobutrazol and ABA resistance tests, surface-sterilised seeds were sown on 0.5% (w/v) agarose supplemented with paclobutrazol (Syngenta; http://www.syngenta-agro.fr/synweb/default.aspx) or ABA at the concentrations indicated. Stratification of seeds at 4°C for 3 d was only carried out for the ABA test. Seeds were then incubated in the same conditions as the dormancy assays for 4 d. Seedlings were scored as resistant if they developed green cotyledons.

### Expression analysis

Rosettes used were from plants that had been grown in soil in the glasshouse for 3 weeks. For dehydrated tissue, rosettes were detached from plants and placed under a laminar flow hood until they were at 75% of their original weight, placed in a plastic tube and then incubated for 4 h in the dark. Total RNA was prepared from frozen plant tissues using Sigma mammalian total RNA kit (Sigma-Aldrich; http://www.sigmaaldrich.com) following the manufacturer's protocol and including an on-column DNase I treatment (RNase-free DNase set; Qiagen). Total RNA (2 µg) was used as a template to synthesize first-strand cDNA using an oligo(dT) 18-mer primer and the SuperScript first-strand synthesis kit (Invitrogen; http://www.invitrogen.com/site/us/en/home.html) according to the manufacturer's instructions. Quantitative real-time PCR reactions were performed using the LightCycler FastStart DNA master SYBR green I kit in a Roche LightCycler 1.0 (Roche; http://www.roche.com). Reactions used 5 µL of 1∶50 diluted sscDNAs in a total volume of 20 µL. Gene-specific primers that had been tested for their efficiency rates and sensitivity on dilution series of cDNAs were as follows: *EF1α-4a*, forward primer, 5′-CTTCTTGCTTTCACCCTTGGTGT-3′, reverse primer, 5′-TGTCAGGGTTGTATCCGACCTT-3′; *RD29B*, forward primer, 5′-CTTCTTGCTTTCACCCTTGGTGT -3′, reverse primer, 5′- TGTCAGGGTTGTATCCGACCTT -3′; *RD22*, forward primer, 5′- CGTCAGGGCTGTTTCCACTGAG -3′, reverse primer, 5′- AGTAGAACACCGCGAATGGGTA -3′; *RD29A*, forward primer, 5′- CCGGTCTCTCTGCTTTCTGG -3′, reverse primer, 5′- CCACTAAGATAGTCTGAAACAGCCGA -3′; *ERD1*, forward primer, 5′- AGAGCTGTGAAGAGGTCCCG-3′, reverse primer, 5′- CCAATCTCAGCATGGATTCTTCCG -3′. The efficiencies of all the primer sets used were almost identical. The reactions were incubated as follows, denaturation of cDNAs and hot start of recombinant *Taq* DNA polymerase at 95°C for 8 min, then 45 cycles of 95°C for 10 s, 59°C for 4 s and 72°C for 9 s. After the final PCR cycle, a fusion curve was generated to verify the specificity of the PCR amplification; samples were heated at 95°C for 1 s before cooling to 65°C for 30 s, followed by an increase to 95°C with a temperature transition rate of 0.1°C s^−1^.

### *Dickeya dadantii* pathogenicity assay

Seeds were sown on soil, stratified for 2 days at 4°C, and incubated at 25°C/20°C (day/night) in short day conditions (8 h photoperiod, 70% RH). After two weeks seedlings were transplanted to individual 49 cm^2^ pots. When plants were six weeks old they were watered abundantly and covered with plastic cloches for 16 h to obtain 100% humidity, which favours infection. For inoculation, the *Dickeya dadantii* 3937 bacterial strain was grown overnight on Lurani-Bertani plates. Cells were then washed from plates and suspended in 50 mM KPO_4_ pH 7 to a concentration of 10^4^ CFU ml^−1^. Inoculation was performed, after wounding one leaf per plant with a needle, by depositing a 5 µl droplet of the bacterial suspension on the wound. The cloches were then replaced in order to maintain a high level of humidity throughout the assay. Resistance to bacterial infection was scored each day for 5 days using the criteria described in Figure 7 and Figure S8.

## Supporting Information

Figure S1 **Confirmation of the absence of xanthine dehydrogenase activity in** ***has1 aba3-1*** **,** ***has2 aba3-1*** **and** ***has3 aba3-1*** **.** Zymogram of total protein extracts from leaves of 21 day-old plants. Band corresponds to coloured product produced by xanthine dehydrogenase activity in the wild-type (WT). Similar results were obtained in 2 independent experiments.
(TIF)

Figure S2 **The** ***has3*** **mutant is not a** ***cyp707a1*** **mutant allele.** A, False colour infrared image of the temperature of drought stressed plants showing that the F_1_ progeny of *cyp707a1-1* crossed with *has3* had colder leaves than the parental genotypes. B, F_1_ progeny of *cyp707a1-1* crossed with *has3* were less resistant to a progressive drought stress then the parental genotypes and wilted.
(TIF)

Figure S3 **Schematic representation of the procedure used for selection of** ***has*** **single mutants.** Chromosomes bearing the *HAS* or *ABA3* loci are represented as purple or blue bars, respectively, and the loci themselves as light blue or black lines, respectively.
(TIF)

Figure S4 **Photosynthesis is not modified in** ***has*** **mutants.** Photosynthesis rates based on leaf net CO_2_ uptake (*A*) were measured as a function of C_i_ (internal CO_2_ molar ratio) under A, ambient oxygen (21%) or B, low oxygen (0.5%) conditions. PPFD during measurements was 800 µmol.m^−2^.s^−1^. WT, wild-type. Error bars represent SE values (n≥3).
(TIF)

Figure S5 **Stomatal density is not modified in** ***has*** **mutants.** Number of stomata measured on abaxial surface, black bars, or adaxial surface, white bars. WT, wild-type. Error bars represent SE values (n≥5). Similar results were obtained in 2 independent experiments.
(TIF)

Figure S6 **Induction of stomatal closure by 10 µM ABA in wild-type and** ***has*** **mutants.** Stomata aperture ratios (width/length) were measured after a 2 h pre-treatment in the light in stomata opening solution followed by a 3 h incubation with or without 10 µM ABA. Error bars represent SE values (n = 40). No significant difference was observed in Student *t*-tests comparing mutant and wild-type at a given ABA concentration. WT, wild-type.
(TIF)

Figure S7 **The** ***has2*** **mutation affects germination characteristics in accord with seed ABA hypersensitivity.** A, Germination of mature, surface-sterilised seeds. The number of seeds with protruding radicles was scored and compared with the total number of seeds sown. B, paclobutrazol resistance of germinating seeds. The number of seeds with green cotyledons was scored and compared with the total number of seeds sown. WT, wild-type. Error bars represent SE values (n = 3).
(TIF)

Figure S8 **Symptom notation for** ***Dickeya dadantii*** **infection.** The scale of infection is denoted by: 0: no symptom; 1: maceration limited to the inoculation point; 2: maceration extends from the infection point; 3: maceration covers half the lamina; 4: maceration spread over the whole lamina; 5: maceration spread over the whole leaf (lamina and petiole).
(TIF)

Table S1 **Primer sequences and number of recombinant F_2_ progeny for markers delimiting the** ***has*** **loci mapping intervals.**
(TIF)

Protocol S1 **Selection of** ***has*** **single mutants.**
(TIF)

Protocol S2 **Photosynthesis measurements.**
(TIF)

Protocol S3 **Stomatal density measurement.**
(TIF)
