# Supplementary figures and images for: New ABA-Hypersensitive Arabidopsis Mutants Are Affected in Loci Mediating Responses to Water Deficit and Dickeya dadantii Infection
Source: PLoS One. 2011 May 25;6(5):e20243. doi: 10.1371/journal.pone.0020243 (PMC3102102; doi:10.1371/journal.pone.0020243)

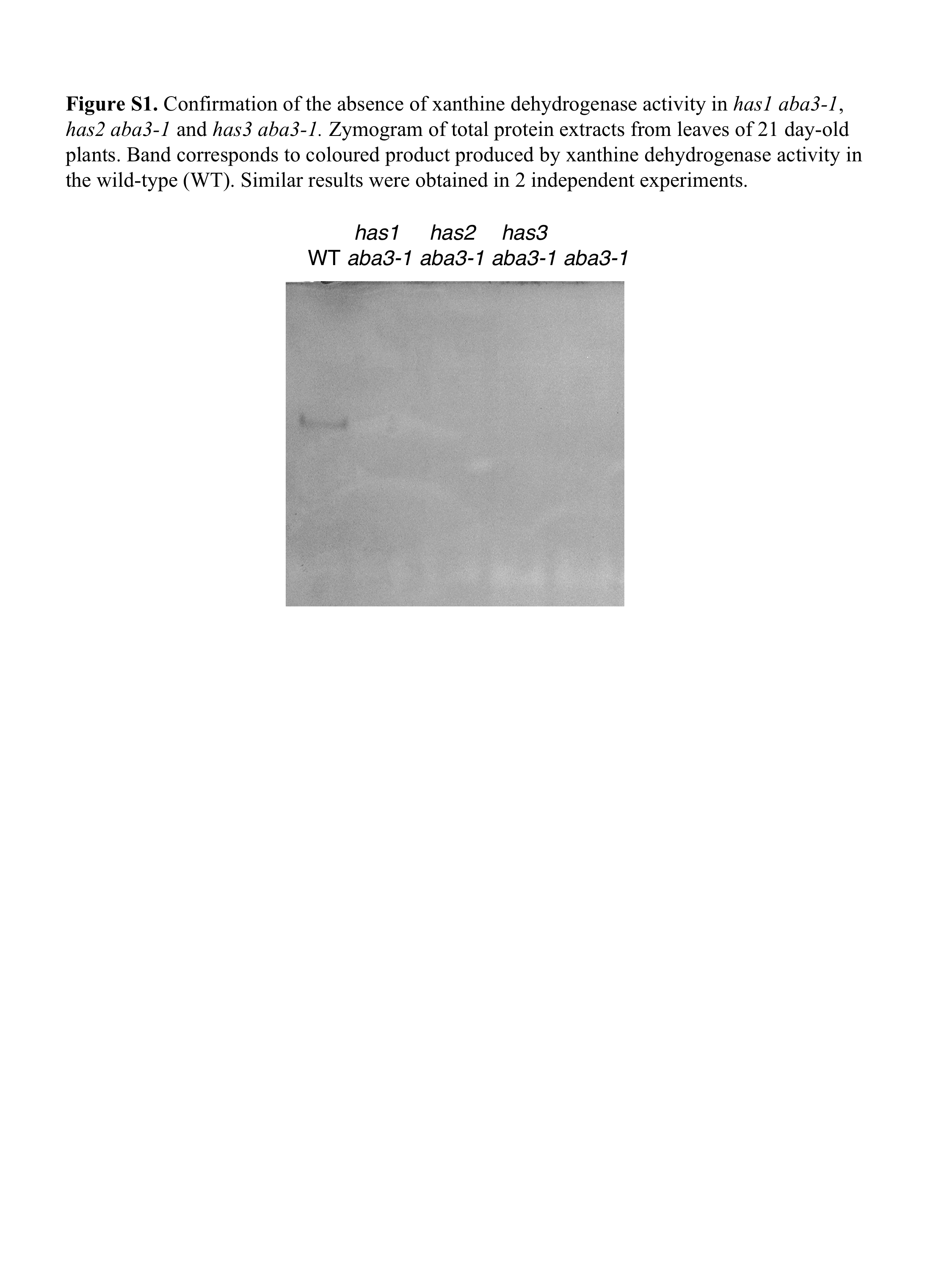

Supplement: Figure S1 — Confirmation of the absence of xanthine dehydrogenase activity in has1 aba3-1 , has2 aba3-1 and has3 aba3-1 . Zymogram of total protein extracts from leaves of 21 day-old plants. Band corresponds to coloured product produced by xanthine dehydrogenase activity in the wild-type (WT). Similar results were obtained in 2 independent experiments. (TIF) [file pone.0020243.s001.tif]

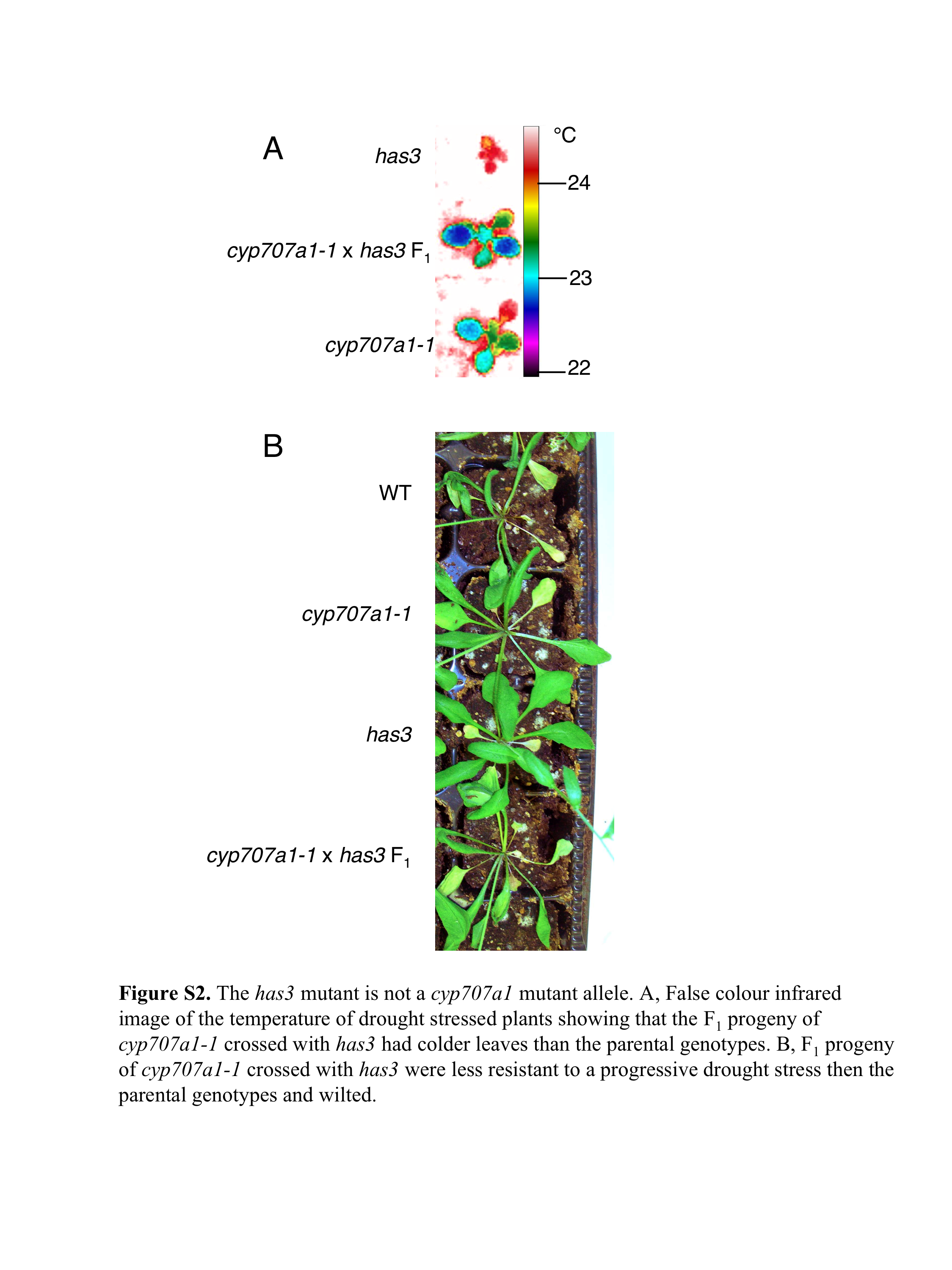

Supplement: Figure S2 — The has3 mutant is not a cyp707a1 mutant allele. A, False colour infrared image of the temperature of drought stressed plants showing that the F1 progeny of cyp707a1-1 crossed with has3 had colder leaves than the parental genotypes. B, F1 progeny of cyp707a1-1 crossed with has3 were less resistant to a progressive drought stress then the parental genotypes and wilted. (TIF) [file pone.0020243.s002.tif]

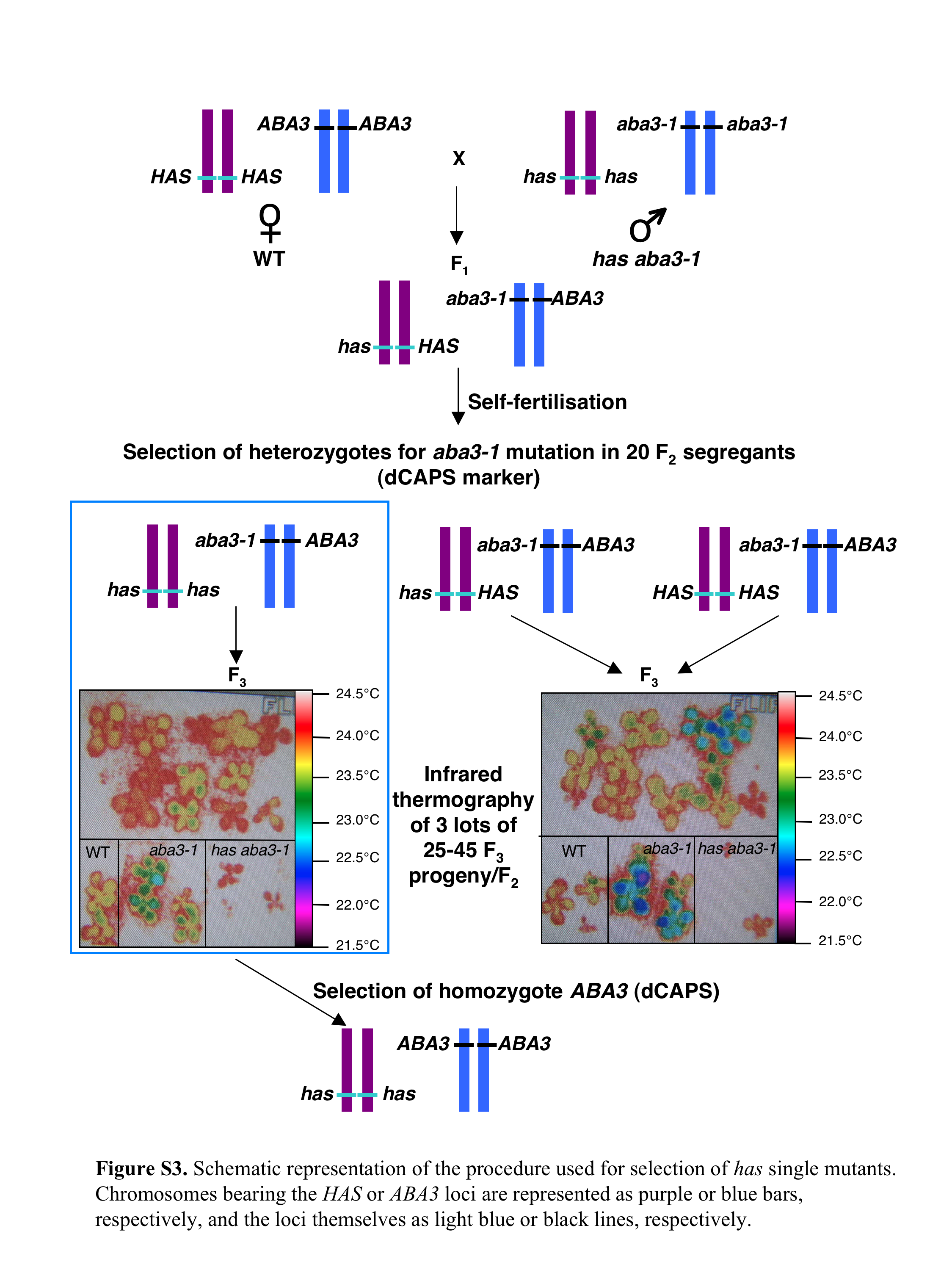

Supplement: Figure S3 — Schematic representation of the procedure used for selection of has single mutants. Chromosomes bearing the HAS or ABA3 loci are represented as purple or blue bars, respectively, and the loci themselves as light blue or black lines, respectively. (TIF) [file pone.0020243.s003.tif]

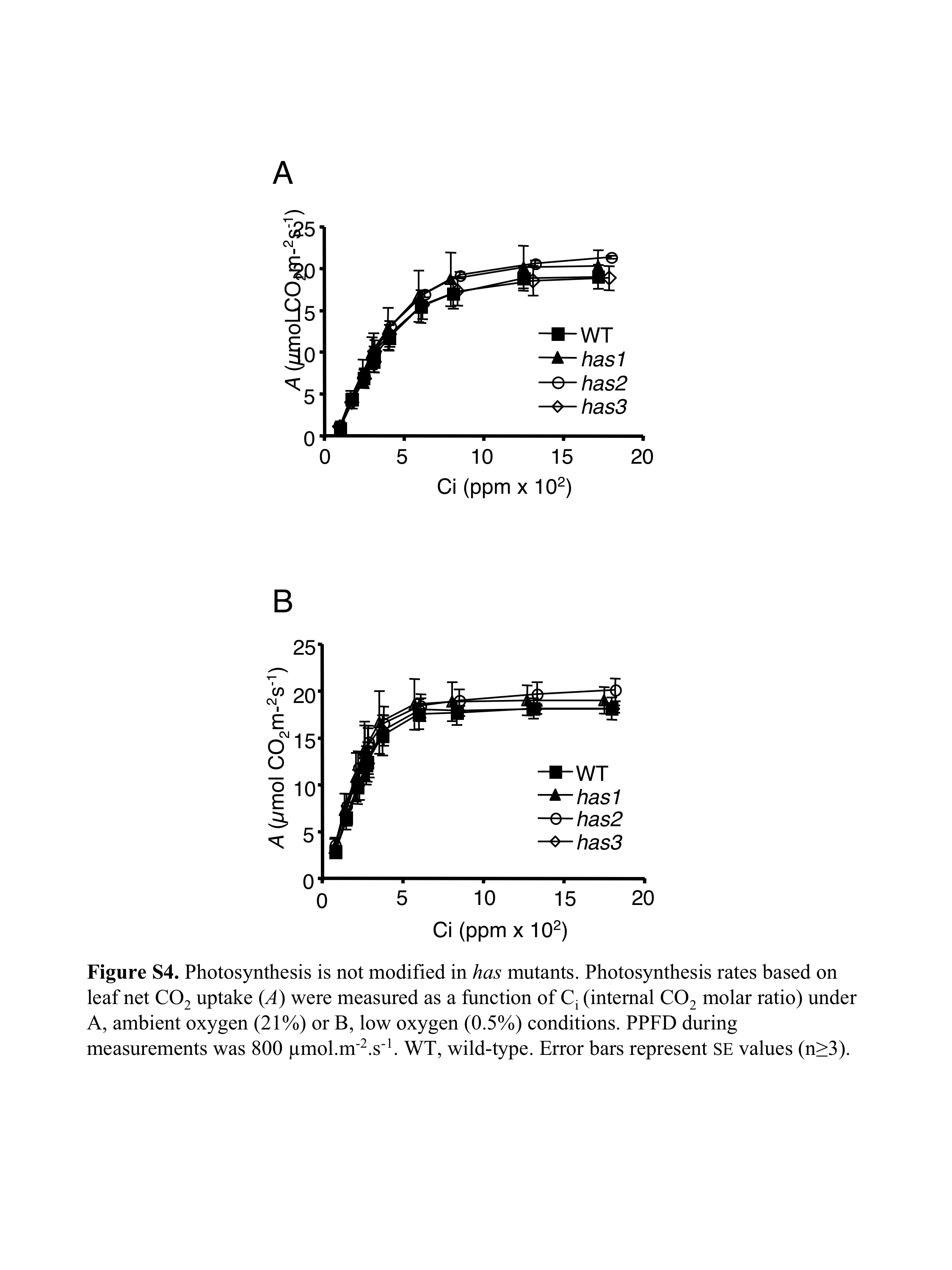

Supplement: Figure S4 — Photosynthesis is not modified in has mutants. Photosynthesis rates based on leaf net CO2 uptake (A) were measured as a function of Ci (internal CO2 molar ratio) under A, ambient oxygen (21%) or B, low oxygen (0.5%) conditions. PPFD during measurements was 800 µmol.m−2.s−1. WT, wild-type. Error bars represent SE values (n≥3). (TIF) [file pone.0020243.s004.tif]

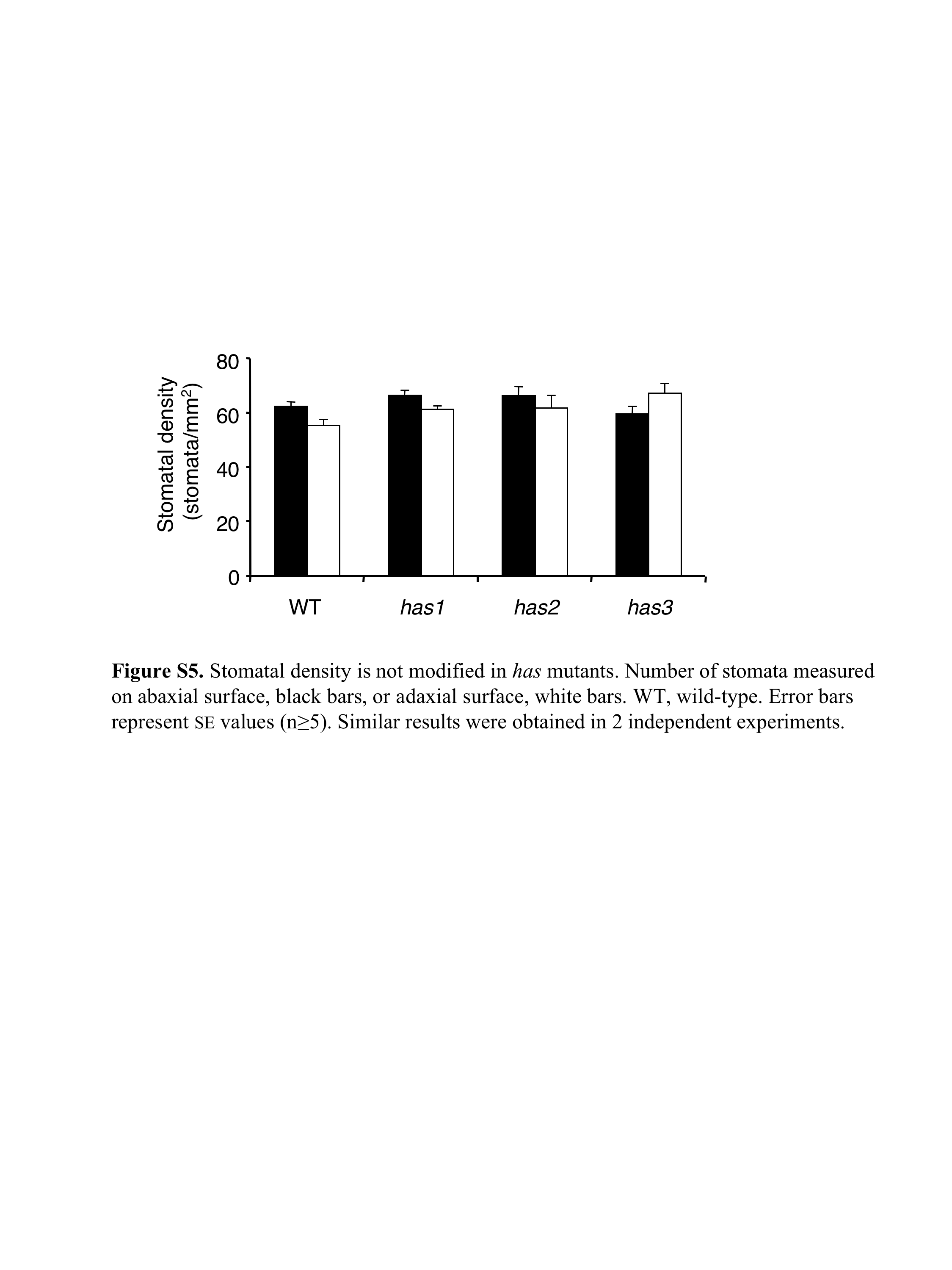

Supplement: Figure S5 — Stomatal density is not modified in has mutants. Number of stomata measured on abaxial surface, black bars, or adaxial surface, white bars. WT, wild-type. Error bars represent SE values (n≥5). Similar results were obtained in 2 independent experiments. (TIF) [file pone.0020243.s005.tif]

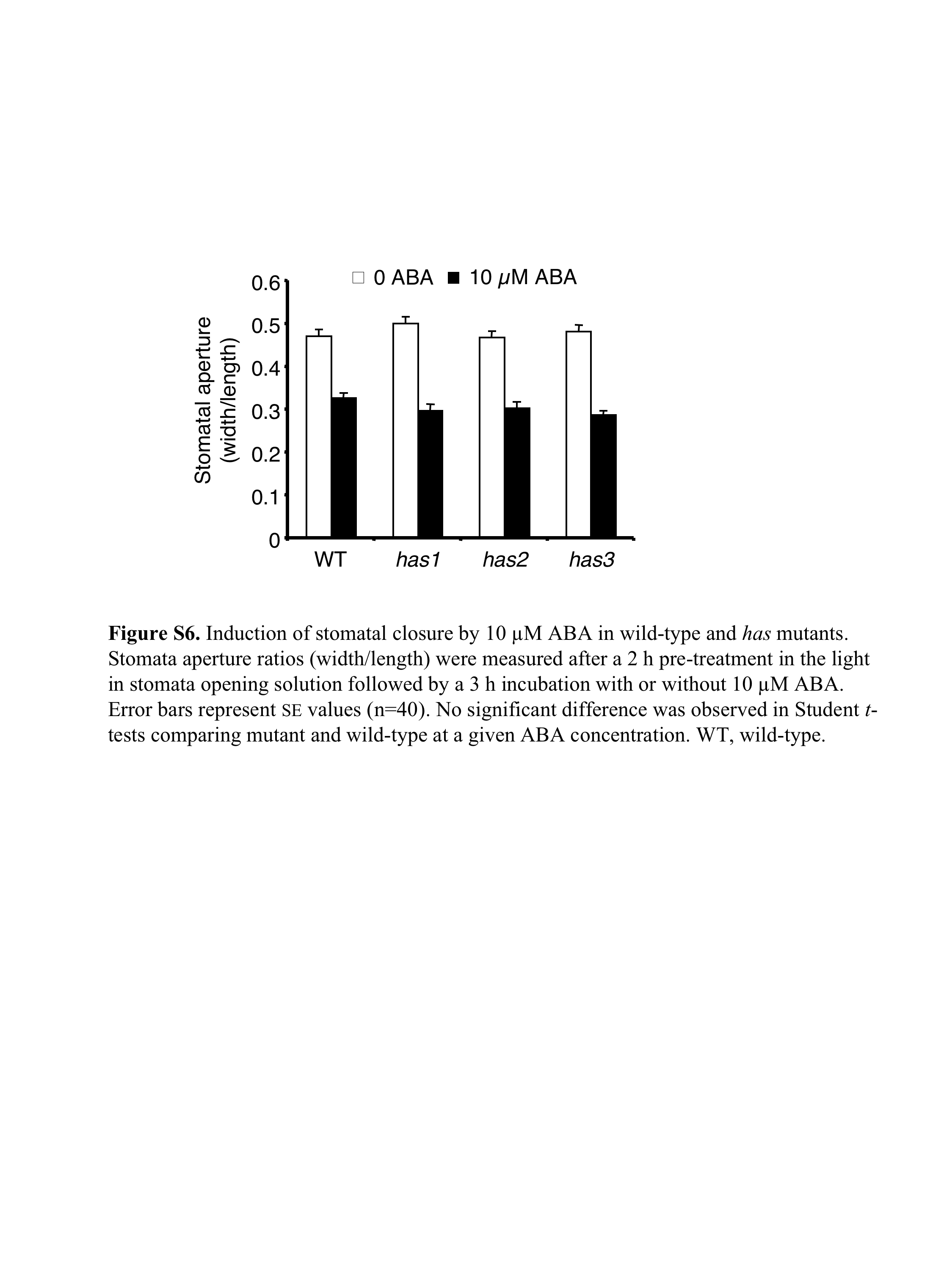

Supplement: Figure S6 — Induction of stomatal closure by 10 µM ABA in wild-type and has mutants. Stomata aperture ratios (width/length) were measured after a 2 h pre-treatment in the light in stomata opening solution followed by a 3 h incubation with or without 10 µM ABA. Error bars represent SE values (n = 40). No significant difference was observed in Student t-tests comparing mutant and wild-type at a given ABA concentration. WT, wild-type. (TIF) [file pone.0020243.s006.tif]

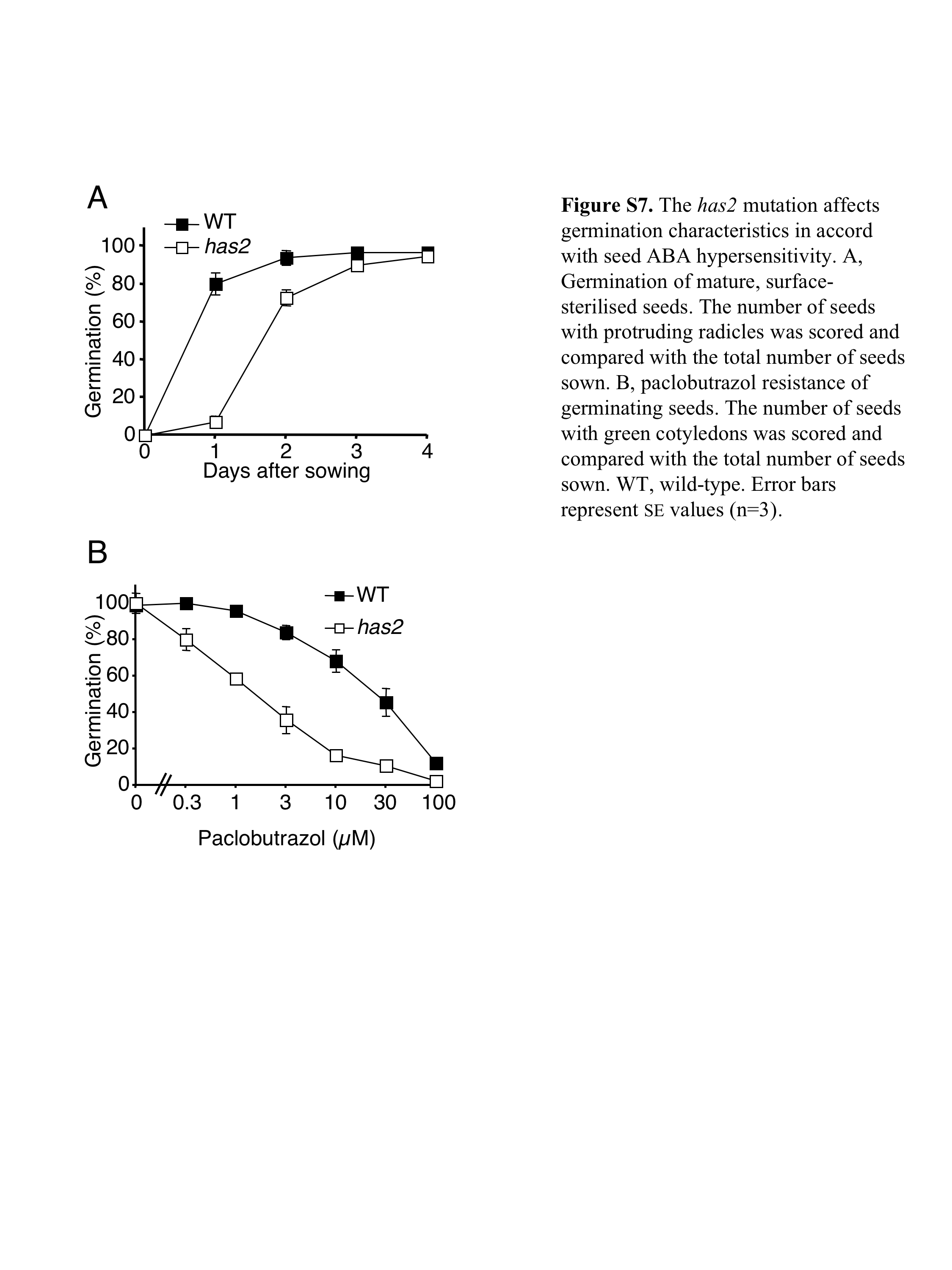

Supplement: Figure S7 — The has2 mutation affects germination characteristics in accord with seed ABA hypersensitivity. A, Germination of mature, surface-sterilised seeds. The number of seeds with protruding radicles was scored and compared with the total number of seeds sown. B, paclobutrazol resistance of germinating seeds. The number of seeds with green cotyledons was scored and compared with the total number of seeds sown. WT, wild-type. Error bars represent SE values (n = 3). (TIF) [file pone.0020243.s007.tif]

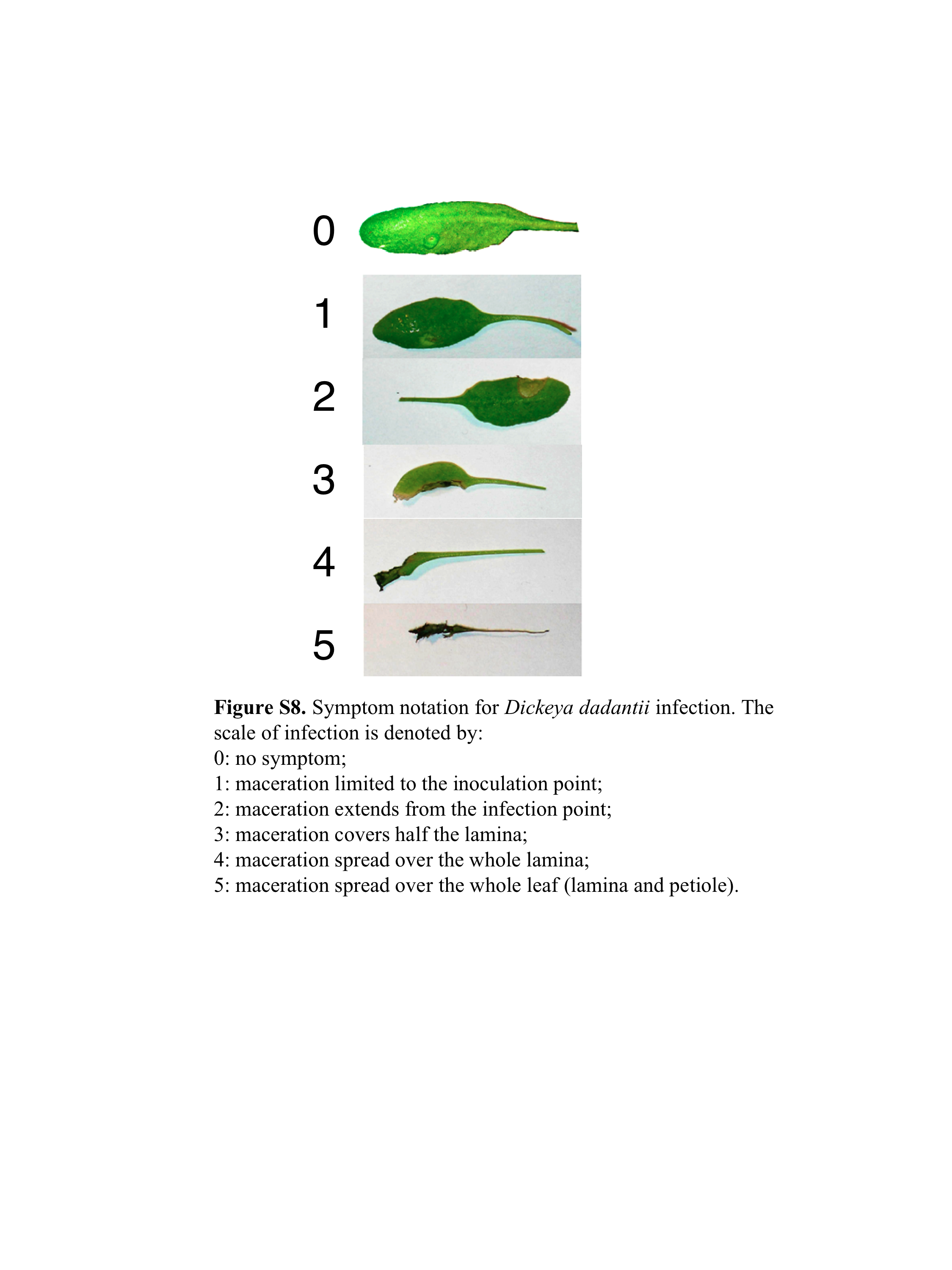

Supplement: Figure S8 — Symptom notation for Dickeya dadantii infection. The scale of infection is denoted by: 0: no symptom; 1: maceration limited to the inoculation point; 2: maceration extends from the infection point; 3: maceration covers half the lamina; 4: maceration spread over the whole lamina; 5: maceration spread over the whole leaf (lamina and petiole). (TIF) [file pone.0020243.s008.tif]

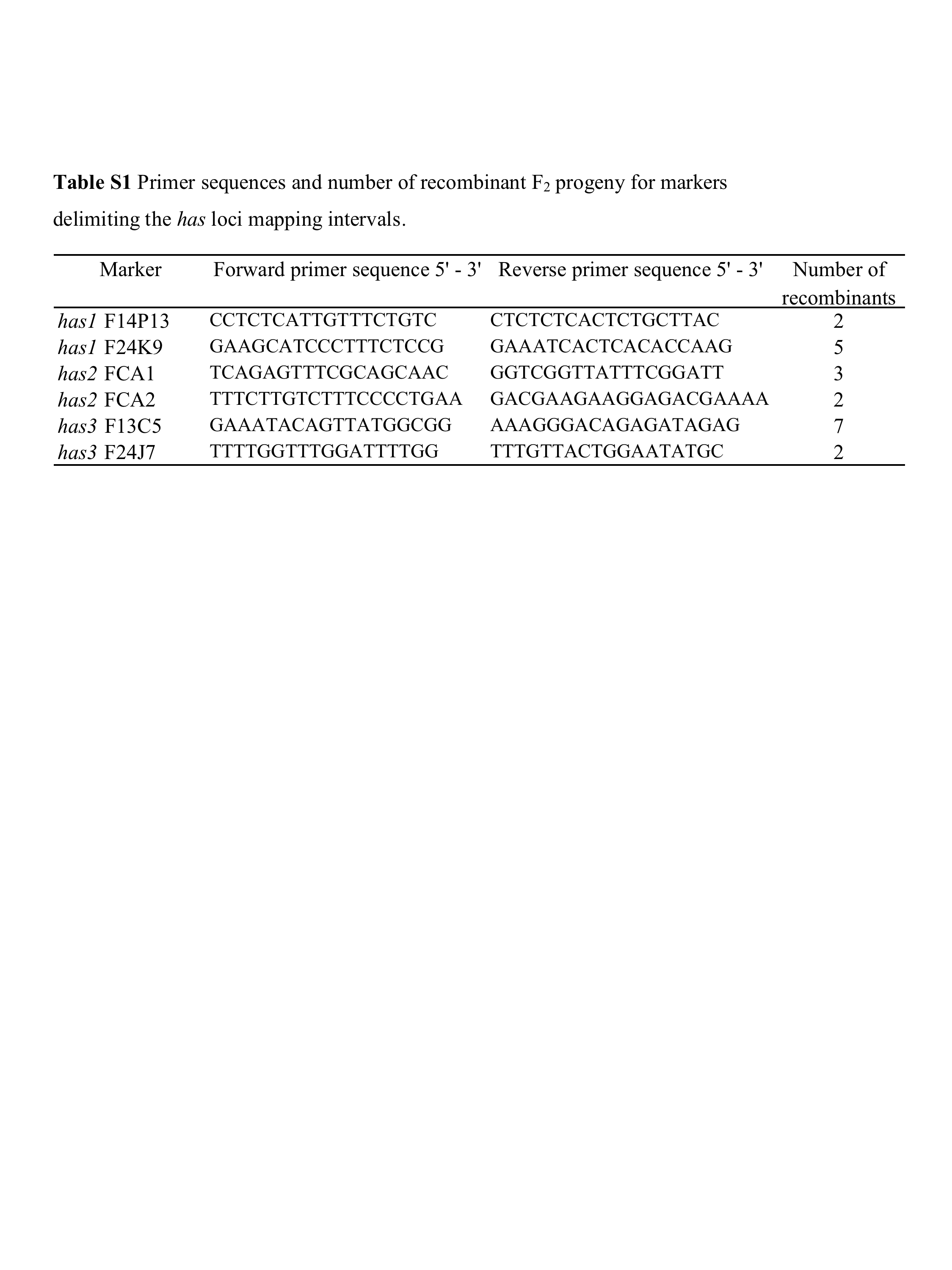

Supplement: Table S1 — Primer sequences and number of recombinant F2 progeny for markers delimiting the has loci mapping intervals. (TIF) [file pone.0020243.s009.tif]

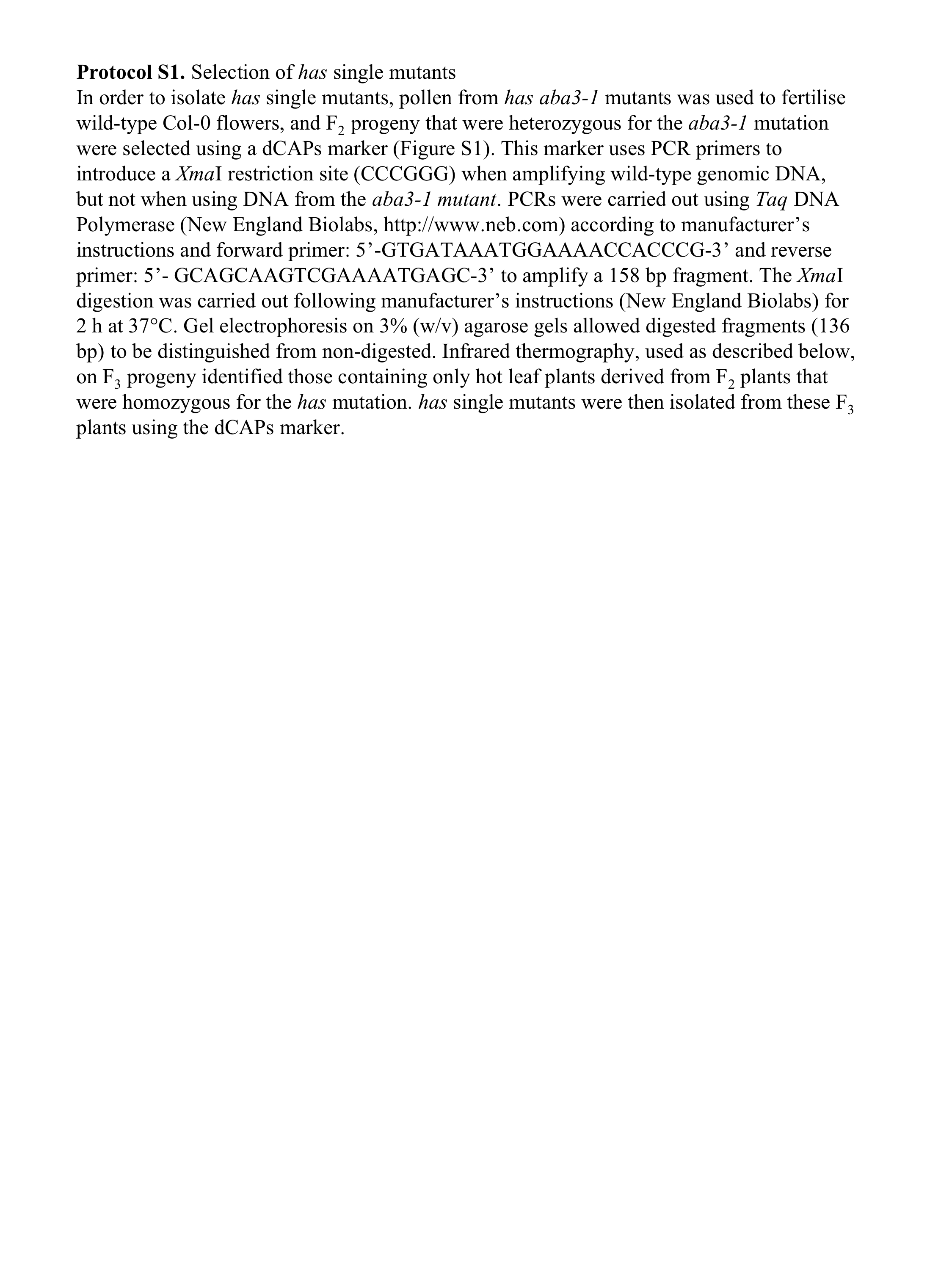

Supplement: Protocol S1 — Selection of has single mutants. (TIF) [file pone.0020243.s010.tif]

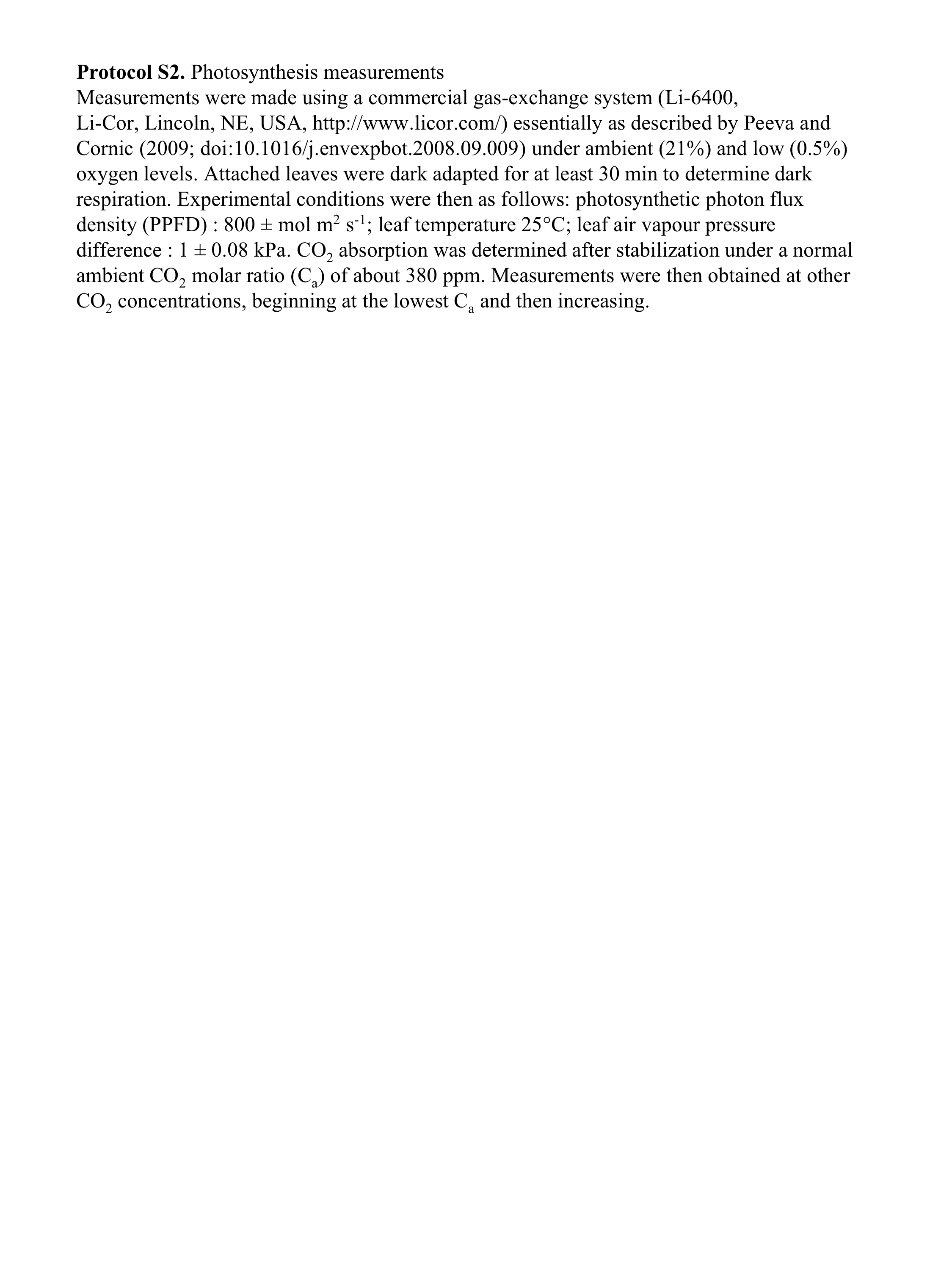

Supplement: Protocol S2 — Photosynthesis measurements. (TIF) [file pone.0020243.s011.tif]

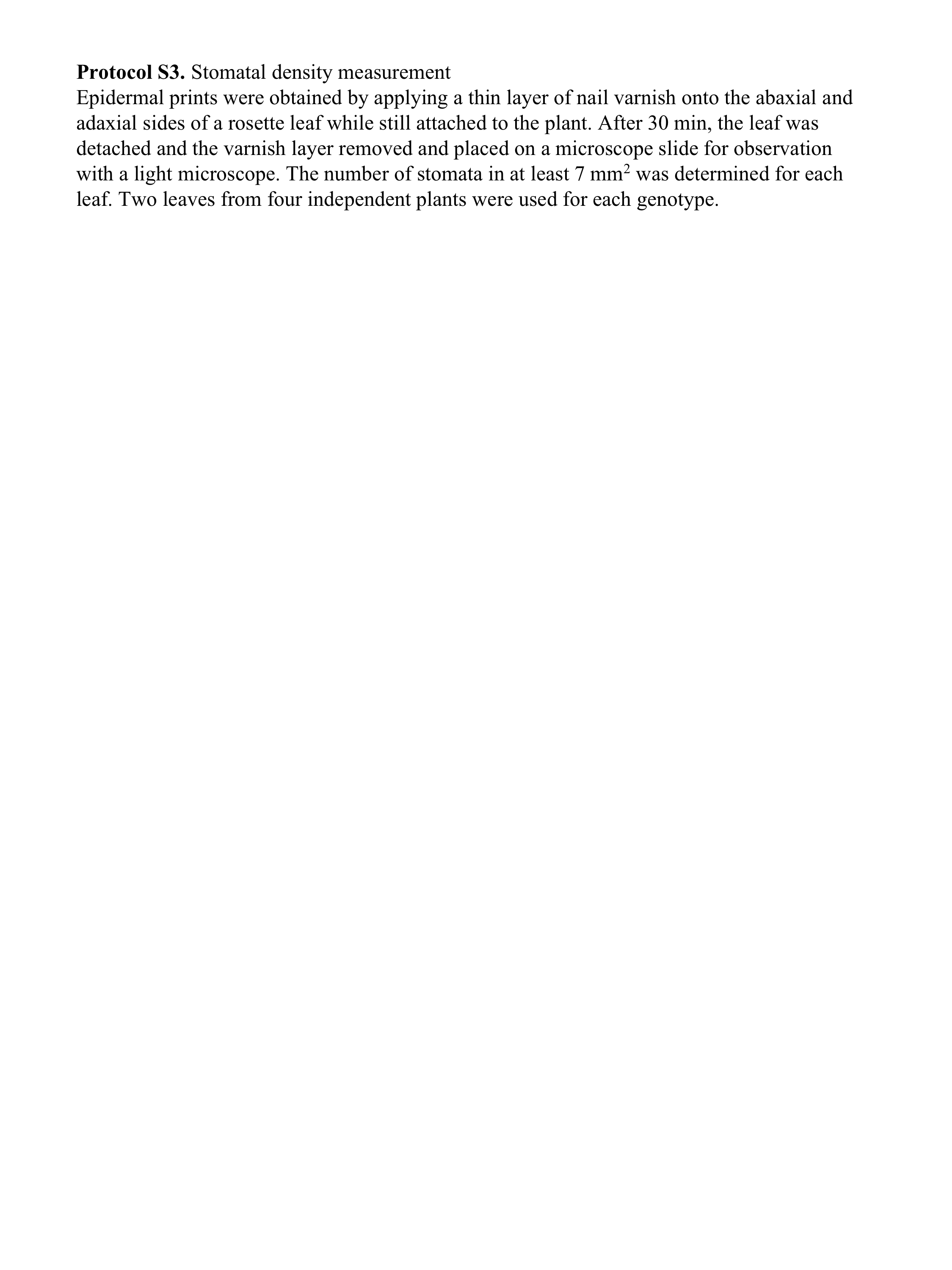

Supplement: Protocol S3 — Stomatal density measurement. (TIF) [file pone.0020243.s012.tif]
